# Co-Supplementation of Baobab Fiber and Arabic Gum Synergistically Modulates the In Vitro Human Gut Microbiome Revealing Complementary and Promising Prebiotic Properties

**DOI:** 10.3390/nu16111570

**Published:** 2024-05-22

**Authors:** Cindy Duysburgh, Marlies Govaert, Damien Guillemet, Massimo Marzorati

**Affiliations:** 1 ProDigest Bv, Technologiepark 82, 9052 Ghent, Belgium; cindy.duysburgh@prodigest.eu (C.D.); marlies.govaert@prodigest.eu (M.G.); 2 Nexira, 129 Chemin de Croisset, 76000 Rouen, France; d.guillemet@nexira.com; 3 Center of Microbial Ecology and Technology (CMET), Ghent University, Coupure Links 653, 9000 Ghent, Belgium

**Keywords:** gut microbiome, prebiotics, baobab fiber, Arabic gum, SHIME^®^ technology, *Christensenellaceae*, *Akkermansiaceae*

## Abstract

Arabic gum, a high molecular weight heteropolysaccharide, is a promising prebiotic candidate as its fermentation occurs more distally in the colon, which is the region where most chronic colonic diseases originate. Baobab fiber could be complementary due to its relatively simple structure, facilitating breakdown in the proximal colon. Therefore, the current study aimed to gain insight into how the human gut microbiota was affected in response to long-term baobab fiber and Arabic gum supplementation when tested individually or as a combination of both, allowing the identification of potential complementary and/or synergetic effects. The validated Simulator of the Human Intestinal Microbial Ecosystem (SHIME^®^), an in vitro gut model simulating the entire human gastrointestinal tract, was used. The microbial metabolic activity was examined, and quantitative 16S-targeted Illumina sequencing was used to monitor the gut microbial composition. Moreover, the effect on the gut microbial metabolome was quantitatively analyzed. Repeated administration of baobab fiber, Arabic gum, and their combination had a significant effect on the metabolic activity, diversity index, and community composition of the microbiome present in the simulated proximal and distal colon with specific impacts on *Bifidobacteriaceae* and *Faecalibacterium prausnitzii*. Despite the lower dosage strategy (2.5 g/day), co-supplementation of both compounds resulted in some specific synergistic prebiotic effects, including a biological activity throughout the entire colon, SCFA synthesis including a synergy on propionate, specifically increasing abundance of *Akkermansiaceae* and *Christensenellaceae* in the distal colon region, and enhancing levels of spermidine and other metabolites of interest (such as serotonin and ProBetaine).

## 1. Introduction

The human microbiota contains up to 10^14^ bacterial cells, which is ten times higher than the number of eukaryotic cells present in the human body. These bacterial cells are able to colonize every body part exposed to the external environment. However, the most heavily colonized organ is the gastrointestinal tract, i.e., 70% of all microbes reside in the intestinal region [1]. These gut microbiota have been reported to have a strong effect on human health and disease. Their presence prevents, amongst others, colonization of the gut by pathogenic species such as *Salmonella* spp. (e.g., by means of nutrient competition or secretion of bacteriocins) [2]. In addition, certain metabolites produced by the intestinal microbiome serve as an important energy source for the host [3]. Butyrate is, for example, recognized as one of the main energy sources for the gut epithelium [4]. The composition and activity of the microbial community have also been related to metabolic syndrome, a group of risk factors including obesity [5]. Gut microbiota can also act as important regulators of the immune system by the production of anti-inflammatory compounds [2]. For example, previous studies reported that the production of butyrate by bacterial species such as *Faecalibacterium prausnitzii* can prevent intestinal inflammation [6]. Finally, the enzymatic activity of some microbial groups proved to result in the conversion of certain food constituents into potentially toxic or carcinogenic compounds related to the occurrence of colorectal cancer (CRC) [7].

The beneficial health effects associated with gut microbiota can be stimulated by the consumption of prebiotics, which have been defined as substrates that are selectively utilized by host microorganisms, conferring a health benefit [8]. The most commonly used prebiotics include fructo-oligosaccharides, inulin, lactulose, and galacto-oligosaccharides [9]. These prebiotics are not digested by the enzymes produced by the human host and end up in the colonic region, where they can be fermented by the gut microbiota [10]. As a result, the composition of the microbial community will be altered due to (i) the difference in fermentation capacity of the different bacterial species, (ii) the antimicrobial activity of some specifically generated metabolites, mainly short-chain fatty acids (SCFA), and (iii) the acid tolerance of the different species [11,12]. These compositional changes can, in turn, also result in a decreased production of certain toxic/carcinogenic compounds such as ammonia [13].

Since most chronic colonic diseases, such as ulcerative colitis and CRC, mainly originate in the distal colon, there is a need for (combinations of) prebiotics that exert biological activity in this specific part of the colon or, preferably, throughout the entire colonic region. However, currently used prebiotics are predominantly fermented in the proximal colon due to their colonization by microbiota with a high saccharolytic potential [14]. The fermentation of Arabic gum (AG), a water-soluble heteropolysaccharide with high molecular weight, is believed to occur mainly in the distal colon due to its rather complex structure and could, therefore, be a promising prebiotic candidate [15,16,17]. This distal fermentation profile was indeed observed by Daguet et al. [18,19] upon long-term administration of AG in an in vitro gut model using fecal inocula of IBS (irritable bowel syndrome) and IBD (irritable bowel disease) donors, respectively. The study of Cherbut et al. [20] also provided evidence for a high digestive tolerance of AG in healthy individuals, i.e., no intestinal symptoms such as flatulence, bloating, abdominal cramps, and diarrhea were reported when dosing up to 30 g/day. Moreover, this repeated intake of AG resulted in increased total lactic acid-producing bacteria and bifidobacteria levels in fecal stools [20]. Similar results were observed in the in vivo study of Calame et al. [21], i.e., a daily dosage of 10 g AG/day increased the amount of bifidobacteria and lactobacilli, which are well recognized for their beneficial health effects related to (i) an increased resistance towards pathogenic colonization and (ii) an increased production of certain SCFA [22]. Other health-promoting effects that have been reported following repeated administration of AG were (i) stimulation of the integrity of the gut wall barrier [18,19], (ii) modulation of gut inflammation [18,19], (iii) transit modulation [23], (iv) kidney health [24], and (v) increased immunity [18,19]. The prebiotic effect of baobab fiber (BF), on the other hand, has not been extensively studied before. Studies investigating its potential health-promoting effects reported strong antioxidative properties linked with its high vitamin C content [25,26]. In addition, preliminary studies revealed metabolic health improvement and gut transit modulation [27,28,29]. Moreover, the study of Foltz et al. [30] revealed a promising prebiotic potential of baobab fruit pulp powder using an in vitro (short-term) fermentation model, i.e., administration of the test product resulted in a stimulated production of acetate, propionate, lactate, and butyrate (to a lower extent) by the gut microbial community of three healthy adult human donors. Finally, an additional benefit of BF might be its potential complementarity with AG, i.e., the relatively simple structure of BF might facilitate breakdown in the proximal part of the colon, leading to a combined prebiotic effect exerted throughout the entire colonic region [31].

In order to systematically examine the prebiotic properties of test substrates on the intestinal microbial community, in vitro approaches are widely used. These in vitro studies range from short-term single-stage colonic incubations (e.g., [32]) to dynamic gut models (e.g., [33,34]), with the latter being able to simulate long-term administration studies under highly controlled and representative intestinal conditions. To mimic the intestinal microbiome as closely as possible, these in vitro gut models rely on fecal inocula from human individuals. While it is generally estimated that the gut microbial community of one individual contains 500 to 1000 different species [35], the collective human microbiome is composed of more than 35,000 bacterial species, which stresses interindividual differences [36]. Therefore, these differences need to be taken into account in in vitro studies, as they can affect the response to dietary interventions. In order to elucidate the potential prebiotic effects of these interventions in sufficient detail, one relies on highly advanced techniques such as 16S rRNA gene sequencing and targeted metabolomic analysis to investigate the effect on microbial metabolic activity and community composition following long-term administration.

Within this study, the main objective was to gain insight into how the human gut microbiota was affected in response to long-term BF and AG supplementation when tested individually or as a combination of both prebiotic compounds in order to investigate potential complementary and/or synergetic effects. For this purpose, the validated Simulator of the Human Intestinal Microbial Ecosystem (SHIME^®^, ProDigest BV and Ghent University, Ghent, Belgium), an in vitro gut model simulating the entire human gastrointestinal tract, was used.

## 2. Materials and Methods

### 2.1. Experimental Design

In the first part of this study, a donor pre-screening test was performed by means of short-term fecal batch fermentations. Three different donors were tested, and it was examined whether the two test products (i.e., AG and BF) affected the metabolic activity of the gut microbiota. The test products were assessed individually and as a combination of both. Moreover, a low and high dosage strategy was investigated. For the combined treatment, in particular, a fiber ratio of 1:1 was used. Samples were taken at different time points, and the metabolic activity was assessed by measuring the pH and the concentration of (i) SCFA, (ii) lactate, (iii) branched SCFA (bCFA), and (iv) ammonium. All conditions were tested in technical replications.

In the second part of this study, a long-term SHIME^®^ experiment was performed using a TripleSHIME^®^ configuration with one donor and one test dose for each individual or combined treatment procedure. The donor was selected based on the results of the short-term fecal batch fermentations. Samples were again collected at different time points in order to assess the effect of the repeated administration of the test products on the metabolic activity and the composition of the gut microbiota. In order to monitor the metabolic activity, the concentrations of (i) SCFA, (ii) lactate, (iii) bSCFA, and (iv) ammonium were determined. For the composition of the gut microbiota, the novel quantitative 16S-targeted Illumina sequencing approach of Vandeputte et al. [37] was used. Moreover, the effect on the gut microbial metabolome was quantitatively analyzed by means of targeted ultra-high-performance liquid chromatography coupled with high-resolution Orbitrap mass spectrometry (UHPLC-LC-MLS/MS).

### 2.2. Chemicals and Test Products

Unless otherwise stated, all chemicals were obtained from Sigma-Aldrich (St. Louis, MO, USA). AG (Inavea pure acacia™) was provided by Nexira (Rouen, France). It is an exudate from the acacia tree, characterized by its content of 90% soluble fiber composed of arabinogalactans hydrocolloid. Monomeric sugar distribution is about 40% galactose, 26% arabinose, 14% rhamnose, and 15% glucuronic acid [38]. The ramified, complex, and heterogeneous polysaccharidic structure is partially linked to low amounts of proteins (about 2%) with a global molecular weight distributed from 20 kDa to 6000 kDa [15,17]. This structure specificity confers resilience to the fiber, a progressive fermentation profile, and high tolerability in the gut [20]. BF was also provided by Nexira and consists of a powder obtained from the baobab fruit pulp. Fibers of the baobab fruit are mainly insoluble (about 64%), and the main classifications contained are cellulose, fructo-oligosaccharides, and pectin (internal analysis). Polysaccharide structure has been described to be a rather small polysaccharide (Mw 53 kDa) in comparison with AG, and it is composed of fructose and glucose [31]. During the short-term fecal batch fermentations, the test products were investigated at two different dosages, i.e., 2.5 g/L and 5 g/L total fiber, with a fiber ratio of 1:1 being applied for the combined treatment. For the long-term SHIME^®^ experiment, only one individual and one combined treatment dose were examined. For the individual treatment, on the one hand, an in vitro daily dosage of 5 g/d BF or AG was applied, corresponding to an in vivo human dose of 10 g/d. For the combined treatment, on the other hand, the in vitro dosage was equal to 2.5 g/d (i.e., 1.25 g/d BF + 1.25 g/d AG), thus corresponding to 5 g/d (i.e., 2.5 g/d BF + 2.5 g/d AG) in vivo. The lower combined test dose, as compared to the individual treatment, was selected in order to examine whether significant effects (as compared to the control period) could still be obtained while optimizing the combined treatment dose. 

### 2.3. Donor Pre-Screening via Short-Term Fecal Batch Fermentations

A donor pre-screening test was performed by means of a short-term fecal batch fermentation experiment simulating the conditions of the proximal large intestine. The appropriate amount of test product was dissolved in water and dosed aseptically to reactors containing 63 mL sugar-depleted nutritional medium (5.2 g/L K_2_HPO_4_ (Chem-lab NV, Zedelgem, Belgium; CL00.1155.1000), 16.3 g/L KH_2_PO_4_ (Chem-lab NV; CL00.1146.1000), 2.0 g/L NaHCO_3_ (Chem-lab NV; CL00.1432.1000), 2.0 g/L yeast extract (Oxoid, Cheshire, UK; LP0021B), 2.0 g/L special peptone (Oxoid; LP0072B), 1.0 g/L mucin (Carl Roth, Karlsruhe, Germany; 8494.7), 0.5 g/L L-cystein (Sigma-Aldrich; 2430-100 GM), and 2.0 mL/L Tween80 (P4780-100 ML)) to obtain a (total) fiber concentration of 2.5 or 5 g/L. Non-sterile anaerobic fecal slurries were prepared from freshly collected feces of three healthy human donors (i.e., donor A, donor B, and donor C) and inoculated at 10% (*v*/*v*) into the respective reactors, resulting in a total reactor volume of 70 mL. A blank was included for each donor to monitor the background activity of the community. For this purpose, only sugar-depleted nutritional medium was provided without the addition of AG and/or BF. All reactors were anaerobically incubated in a shaking incubator (90 rpm) at 37 °C for 48 h. Two technical replicates were performed for each treatment (and blank) procedure. As a consequence, 14 incubation experiments were performed for each donor or 42 experiments in total (i.e., 7 test conditions × 2 replicates × 3 donors, with blank, 2.5 and 5 g/L baobab fiber (BF), 2.5 and 5 g/L Arabic gum (AG), and 2.5 and 5 g/L BF + AG as test conditions).

### 2.4. Simulator of the Human Intestinal Microbial Ecosystem (SHIME^®^)

The reactor setup used to simulate the human gastrointestinal tract was derived from the SHIME^®^ (ProDigest BV and Ghent University, Ghent, Belgium) as described by Molly et al. [33]. However, the SHIME^®^ setup was adapted from a single SHIME^®^ configuration (including one SHIME^®^ arm) to a TripleSHIME^®^ configuration (including three SHIME^®^ arms) in order to allow a parallel comparison between the three different treatment conditions. Each arm of the TripleSHIME^®^ configuration consisted of a succession of three reactors, with each of them representing a different region of the human gastrointestinal tract. The first reactor was used to simulate the upper gastrointestinal tract, followed by the subsequent simulation of the gastric and small intestinal phases. The second and third reactors were used to simulate the proximal colon (PC) and the distal colon (DC), respectively. The PC reactor, on the one hand, was operated at pH 5.6–5.9 and had a retention time of 20 h. The DC reactor, on the other hand, was operated at pH 6.6–6.9, and the retention time was set at 32 h. Inoculum preparation, temperature settings, feeding regime, and reactor feed composition were adopted from Possemiers et al. [39]. Therefore, the authors refer to this specific study for more detailed information concerning these experimental aspects. 

Upon inoculation of the PC and DC reactors with the fecal inoculum from the selected donor, a two-week stabilization period was initiated. This stabilization period allows the fecal microbiota to differentiate in the colonic reactors depending on the local environmental conditions (e.g., difference in pH). Subsequently, the baseline microbial community composition and activity were determined in the PC and DC reactors during a two-week control period, during which stability and reproducibility of the model were confirmed, reaching an average of 93.1% and 91.0%, respectively. Finally, a three-week treatment period of repeated daily administration of the test product(s) was incorporated to examine the effect of the treatment on the activity and the composition of the gut microbiota. The test products (i.e., AG and/or BF) were added to the SHIME nutritional medium and administered during each feeding cycle to obtain the corresponding daily doses. Thus, the test products were first transferred to the upper gastrointestinal tract region before reaching the colon vessels. It should be noted that SHIME nutritional medium (containing 1.2 g/L Arabic gum, 2.0 g/L pectin, 0.5 g/L xylan, 4 g/L starch, 0.4 g/L glucose, 3 g/L yeast extract, 1 g/L special peptone, 3 g/L mucin, and 0.5 g/L L-Cystein, ProDigest BV) was administered during both the stabilization and control period in order to allow differentiation of the gut microbial community within the proximal and distal colonic region, similar as what would occur in an in vivo situation. However, the SHIME nutritional medium was supplemented with AG and/or BF during the treatment period in order to determine whether AG and/or BF had an effect on the gut microbial activity and composition. Thus, the results of the treatment period were always compared to the results of the corresponding control period.

### 2.5. Analysis of the Microbial Metabolic Activity

For the short-term fecal batch fermentation experiments, the metabolic activity of the microbial community present in each reactor was examined at three different time points, i.e., following 0, 24, and 48 h of incubation. In order to characterize the metabolic activity, pH values were measured using a Senseline pH meter F410 (ProSense, Oosterhout, The Netherlands). Moreover, the concentration of different metabolites was assessed. The concentration of SCFA, including acetate, propionate, butyrate, and bCFA (i.e., the sum of isobutyrate, isovalerate, and isocaproate), was determined according to the procedure of De Weirdt et al. [40]. Moreover, also the total concentration of all identified linear and branched SCFA was calculated, i.e., by making the sum of acetate, propionate, isobutyrate, butyrate, isovalerate, valerate, isocaproate, and caproate concentrations. The lactate levels were assessed using a commercially available enzymatic assay kit (R-Biopharm Nederland B.V., Arnhem, The Netherlands; E8240) according to the manufacturer’s instructions. Finally, ammonium levels were determined, as previously reported by Duysburgh et al. [41]. Ideally, administration of the fibers should result in an alteration of the environmental pH and an increased production of lactate and SCFA, with propionate and butyrate being the most important metabolites due to their health-promoting effect.

During the long-term SHIME^®^ experiment, the effect of the test products on the metabolic activity of the gut microbiota was also assessed based on the concentration of (i) SCFA (acetate, propionate, butyrate, bCFA, and total SCFA), (ii) lactate, and (iii) ammonium at different time points. For each colonic reactor (i.e., PC and DC), samples were collected three times per week during the entire control and treatment period.

### 2.6. Microbial Community Analysis through Quantitative 16S-Targeted Illumina Sequencing

In order to determine the microbial community composition in the long-term SHIME^®^ experiment, samples were collected three times per week during the final week of the control and treatment period from the PC and DC reactors. To obtain proportional abundances at different phylogenetic levels (phylum, family, and OTU), 16S-targeted Illumina sequencing (LGC Genomics GmbH, Berlin, Germany) was applied using the procedure of Props et al. [42]. Briefly, library preparation and sequencing were performed on an Illumina Miseq platform with v3 chemistry. The V3-V4 hypervariable regions of the 16S rRNA gene were amplified using primers 341F and 785Rmod (5′-CCT ACG GGN GGG WGC AG-3′ and 5′-GAC TAC HVG GGT ATC TAA KCC-3′, respectively). As described in Schloss and Westcott [43] and Kozich et al. [44], the 16S-targeted sequencing analysis was adapted from the MiSeq protocol for read assembly and clean-up using the mothur software (v. 1.39.5). The different steps applied within this procedure were the following: (i) assembling of reads into contigs, (ii) alignment-based quality filtering performed by alignment to the mothur-reconstructed SILVA SEED alignment (v. 123), (iii) removal of chimeras, (iv) assignment of taxonomy via a naive Bayesian classifier [45] and RDP release 14 [46], and (v) clustering of contigs into OTUs at 97% sequence similarity. In addition, sequences classified as Eukaryota, Archaea, Chloroplasts, Mitochondria, and non-classified sequences were removed. For each OTU, representative sequences were selected as the most abundant sequence within that OTU. Furthermore, the obtained proportional abundances were used to calculate the reciprocal Simpson diversity index. This value is a measure of both the diversity and the evenness of the population and was calculated by the formula presented in Equation (1).

(1) $$Reciprocal\;Simpson\;Diversity\;Index=\frac{1}{\sum_{i=1}^{n}p{roportional\;abundance}_{i}^{2}}$$

with *n* = the total number of OTUs detected in one sample.

From each colonic reactor, samples were also collected three times per week during the final control and treatment week for enumeration of the bacterial cells via flow cytometry. A ten-fold dilution series was initially prepared in a phosphate-buffered saline (PBS) solution. Assessment of the viable, non-viable, and total population of the microbial community was performed by staining the appropriate dilutions with SYTO 24 (0.1 mM; Ex/Em: 480/500 nm; Life Technologies Europe B.V., Bleiswijk, The Netherlands; S7559) and propidium iodide (0.2 mM; Ex/Em: 490/635 nm; Fisher Scientific B.V., Merelbeke, Belgium; 11599296). Samples were analyzed on a BD FACSVerse (BD Biosciences, Erembodegem, Belgium). The samples were run using a high flow rate, and bacterial cells were separated from medium debris and signal noise by applying a threshold level of 200 on the SYTO channel. Proper parent and daughter gates were set to determine all populations. The data were analyzed using the FlowJo software (version 10.5.2), and the results were obtained as log (cells/mL).

By combining the proportional abundance values obtained by the 16S-targeted Illumina sequencing method and the total amount of bacterial cells determined through flow cytometry, quantitative abundances of the different taxonomic entities inside the reactors could be determined. As a result, a potential community shift following the administration of the test products could be mapped in large detail.

### 2.7. Evolution of the Gut Microbiome Activity Using Targeted Metabolomics

The dynamics of the metabolic patterns of the gut microbiome were analyzed during the last week of both the control and treatment period (three samples/week) of the long-term SHIME^®^ experiment, and this was for both colonic regions and for each test condition. The metabolomic profile was quantitatively analyzed (Biocrates Life Sciences AG, Innsbruck, Austria) by means of targeted ultra-high-performance liquid chromatography coupled with high-resolution Orbitrap mass spectrometry (UHPLC-LC-MLS/MS). Moreover, the Biocrates MxP^®^ Quant 500 kit (Biocrates, Innsbruck, Austria; 21094.12) was applied according to the manufacturer’s instructions in order to analyze approximately 500 microbiome-related metabolites. A custom-made UHPLC column, based on a ZORBAX Eclipse XDB-C18 column (Agilent Technologies, Santa Clara, CA, USA), and a Triple Quad mass spectrometer Xevo^®^ TQ-S (Waters, Milford, MA, USA) were used, and each identified metabolite was categorized into a different metabolite category as summarized in Table S1.

### 2.8. Data Processing and Statistical Analysis

Principle component analysis (PCA) was applied as a tool to aid the selection of the most appropriate donor following the short-term fecal batch fermentations. The metabolic activity results obtained from the incubations (i.e., pH, acetate, propionate, butyrate, bCFA, and total SCFA) were thereto normalized and uploaded to an online software program, i.e., ClustVis 1.0. The normalized results were included for all donors (i.e., donor A, donor B, and donor C) and all test conditions (i.e., blank, 2.5 or 5 g/L AG, 2.5 or 5 g/L BF, and 2.5 or 5 g/L BF + AG). Relative changes between 0 and 48 h of incubation were applied, and the normalized values were also calculated for an ‘average donor’ in order to identify strong donor-specific effects. The metabolic activity results were based on three biological replicates (=three donors) and two technical replicates. A confidence level of 70% was selected.

With respect to the long-term SHIME^®^ experiment, metabolic activity and microbial community composition results (*n* = 3) were analyzed by means of a two-tailed homoscedastic t-test. This type of statistical analysis was used to compare (i) the corresponding control and treatment periods for a specific colonic region and (ii) the PC and DC region during the control period. It should be stressed that statistical tests regarding the microbial community composition were performed on the log_10_-transformed data (to make them normally distributed). Differences were considered statistically significant if the *p*-value was less than 0.05. For the reciprocal Simpson diversity index, statistical analysis was performed via a fixed linear model analysis of variance (i.e., one-way ANOVA; *p* < 0.05) after verification of the normality of the log-transformed data by means of a Shapiro–Wilk test.

With respect to the targeted metabolomics performed on samples from the long-term SHIME^®^ experiment (*n* = 3), outlier analysis, cleaning, imputation, and log_2_-transformation of the data was performed prior to statistical analysis. Outlier analysis was performed by means of the interquartile range (IQR) method developed by Tukey [47]. For this purpose, the data were divided into four groups of equal size, setting three thresholds, and the threshold values defining the groups were calculated. Then, the IQR was calculated as the range between the first and third threshold, and any data point that was more than 1.5 times the IQR above the upper or more than 1.5 times the IQR below the lower threshold was considered an outlying value. Samples with markedly above-average outlier numbers were excluded if a biological reason for the anomaly was unlikely. Data cleaning was performed in order to exclude metabolites of which concentration values were missing or below the limit of detection (LOD). As a consequence, analytes were only included for further statistical analyses if at least 80% of valid values above the LOD per analyte were available in at least one group of the samples. After cleaning, the dataset still contained 164 metabolites. Missing value imputation was used to logically replace missing values with non-zero values while maintaining the overall data structure, according to Kooperberg and Stone [48]. Then, the dataset was further processed by log_2_-transformation to make assumptions about statistical tests (e.g., symmetric distribution, correction of heteroscedasticity, and skewness of the data) and to improve interpretability and visualization. Moreover, the MetIDQ™ MetaboINDICATOR module was used to calculate sums and ratios of selected metabolites or metabolic classes. Next, the normality of the log-transformed data was checked by means of a Shapiro–Wilk test and statistical analysis was performed via a fixed linear model analysis of variance (i.e., one-way ANOVA). The significance level was set to 0.05, and *p*-values were calculated. In order to control the false discovery rate (FDR) in multiple comparisons, FDR-adjusted *p*-values (q-values) were calculated using the Benjamini and Hochberg method [49]. A q-value of <0.05 was considered statistically significant. This type of analysis was performed for the individual components as well as for the sums and ratios obtained while using the MetaboINDICATOR module. Moreover, for each colonic region, separate ANOVA tests were performed to elucidate potential differences between (i) corresponding control and treatment periods and (ii) the different treatments. Finally, separate heatmaps were prepared for each test product (i.e., BF, AG, and BF + AG) and each colonic region (i.e., PC and DC) to visualize the metabolomic results. Each row represents a different metabolite, or sum/ratio of metabolites, while each column represents a different sample with a specific color coding being applied, ranging from red to blue. For some microbiome-related metabolites of interest, box plots were prepared as well. These box plots include the minimum, maximum, and median values for each test condition during the control and treatment period.

Finally, to investigate potential associations between the metabolites and the metagenomic taxa, the metabolomics, microbial metabolic activity, and microbial community datasets were integrated using non-square correlation matrices. Fold changes were calculated by comparing each treatment to their corresponding control, using the data collected during the final control and treatment week (*n* = 3). Values below the limit of detection (LOD) were replaced by ½ of the LOD. As an additional pre-processing step, metabolites and taxa were excluded from analysis if the number of LODs exceeded half of the total observations per investigated group. Fold changes were subsequently normalized via log_10_-transformation. Pearson correlation coefficients and t-test-derived *p*-values were calculated in R (R version 4.3.1 (2023-06-16 ucrt)) [50], comparing each metabolite and microbial family or phylum for each treatment per vessel [51]. Then, non-square correlation matrices were generated using the corrplot package [52] to visualize the pairwise association between the metabolites and metagenomic families or phyla. 

## 3. Results

### 3.1. Short-Term Fecal Batch Fermentation Experiment

The results of the short-term fecal batch fermentation experiment (Supplementary Figure S1) indicated that each of the donors could have been selected for the long-term SHIME^®^ experiment. Nevertheless, it opted to select donor A since this specific donor resulted in (i) the strongest pH decrease, (ii) the highest levels of total SCFA, acetate, and propionate, and (iii) the lowest levels of bCFA and ammonium (individual results not shown).

### 3.2. Long-Term SHIME^®^ Experiment

#### 3.2.1. The SHIME^®^ Model as a Tool for the In Vitro Simulation of the Gut Microbiota Present in the Different Colon Regions

The microbial community composition of the proximal colon (PC) and distal colon (DC) reactors during the long-term SHIME^®^ experiment was initially examined at the end of the control period in order to verify whether the difference in experimental conditions (i.e., pH and retention time) clearly resulted in a differentiation between both colonic regions. Quantitative 16S-targeted Illumina sequencing was used to determine the average levels (log (cells/mL)) of the different bacterial families encountered in the PC and DC region (Table S2). It was observed that the main phyla in the microbial community of the donor prior to administration of BF and/or AG included Actinobacteria, Bacteroidetes, Firmicutes, and Proteobacteria. However, for the majority of the bacterial families, significant differences were clearly observed between the two distinct colonic regions. Several bacterial families such as *Christensenellaceae* and *Ruminococcaceae* from the Firmicutes phylum, *Barnesiellaceae*, and *Rikenellaceae* from the Bacteriodetes phylum, and *Bifidobacteriaceae* from the Actinobacteria phylum specifically colonized the DC region. Moreover, the low abundance of Verrucomicrobia phylum was specifically detected in this colonic region. Other bacterial families, such as *Bacillaceae* from the Firmicutes phylum and *Enterobacteriaceae* from the Proteobacteria phylum, were mainly present in the PC region. Nevertheless, the highest cell density was observed overall in the DC region.

#### 3.2.2. Metabolic Activity

**Figure 1:**
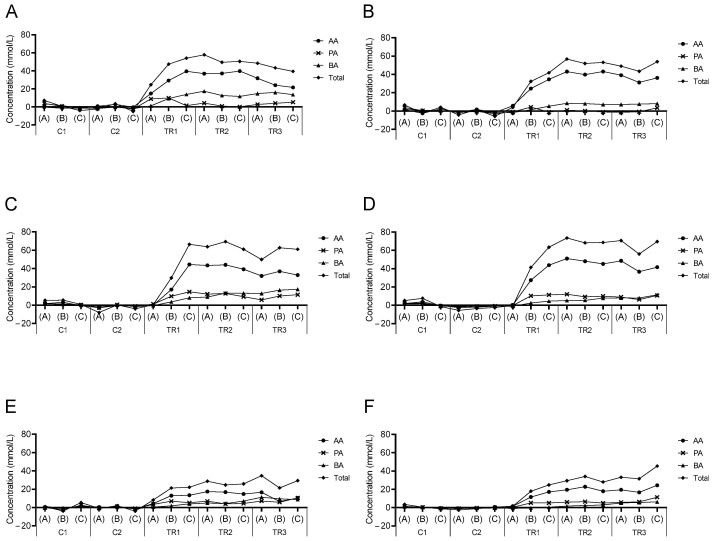
Normalized SCFA profiles during the long-term SHIME^®^ experiment. Normalized kinetic values (mmol/L) of acetate (AA), propionate (PA), butyrate (BA), and total SCFA associated with treatment with baobab fiber (BF; (**A**,**B**)), Arabic gum (AG; (**C**,**D**)), and the combination of both (BF + AG; (**E**,**F**)) in the proximal (PC; (**A**,**C**,**E**)) and distal colon (DC; (**B**,**D**,**F**)) region. Samples were taken during two control (C1–C2) and three treatment (TR1–TR3) weeks, with each week three samples (A–C) being collected and presented as single data points. Average concentrations obtained during the control period (i.e., based on all six samples collected during the first (C1) and second (C2) week) were thereto subtracted from the corresponding concentration obtained at a specific time point.

The metabolic activity of the gut microbiota during the long-term SHIME^®^ experiment was assessed three times per week during the control and treatment period for each colonic vessel. First, upon assessment of the normalized kinetic SCFA profiles (Figure 1), a sudden increase in total SCFA concentrations was observed at the start of the treatment period for all experimental test conditions, with the strongest increases upon supplementation of the individual test compounds. The total SCFA and acetate concentrations instantly increased, while propionate and butyrate levels enhanced more gradually. Moreover, the propionate concentration often declined at one point during the treatment period, while the butyrate concentration increased at the same time. For the BF treatment, similar final increases in total SCFA were observed in both colonic regions. For the AG treatment and the combined treatment (BF + AG), on the other hand, the increase in total SCFA was slightly enhanced in the DC region compared to the PC region. Furthermore, upon calculating the average levels of the measured metabolic parameters during each experimental period (Table 1), it was noticed that acetate, propionate, butyrate, and lactate concentrations were significantly higher during the treatment period compared to the control period. This was valid for each treatment condition and each colonic region with only one exception, i.e., for propionate in the DC region following BF supplementation, for which no significant differences were observed between the experimental periods. Finally, treatment with the different test products consistently reduced bCFA and ammonium levels in both colonic regions.

#### 3.2.3. Microbial Community Composition

**Figure 2:**
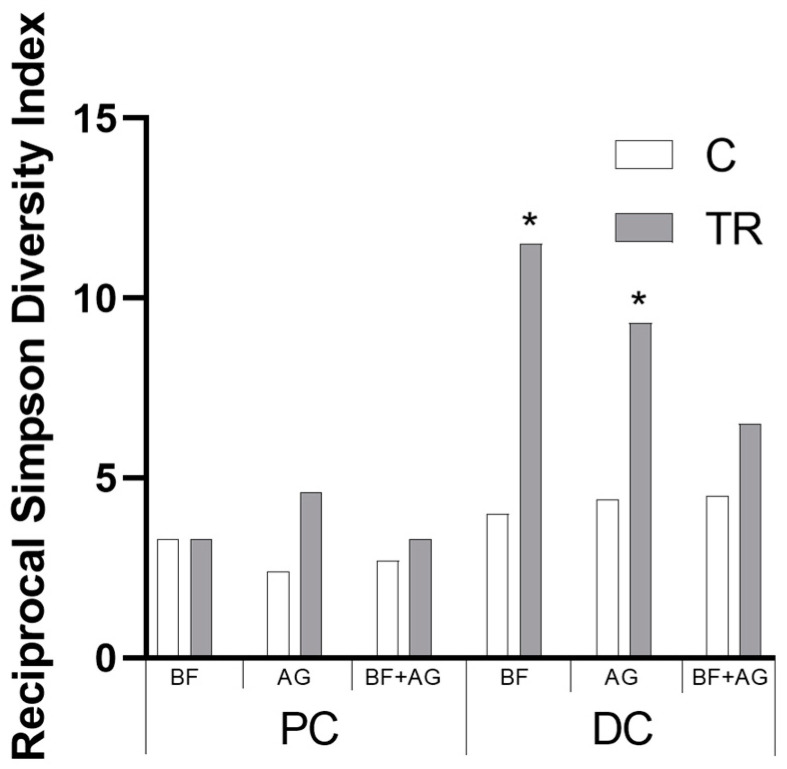
Microbial community diversity during the long-term SHIME^®^ experiment. Average reciprocal Simpson diversity index of the proximal colon (PC) and distal colon (DC) upon treatment with baobab fiber (BF), AG (Arabic gum), and their combination (BF + AG) at different time points during the study, i.e., at the end of the control (C) and treatment period (TR) (*n* = 3). Significant differences between the corresponding control and treatment periods have been indicated by means of ‘*’ (*p* < 0.05).

The microbial community composition was determined both at the end of the control and treatment period for all treatment conditions and both colonic regions using 16S-targeted Illumina sequencing and flow cytometry. First, the reciprocal Simpson diversity index was calculated as a measure of both the diversity and evenness of the microbial community (Figure 2). Repeated administration of the different test products tended to increase the reciprocal Simpson diversity index and thus the population diversity (except in the PC upon BF supplementation), reaching significance in the DC region upon administration of the individual test products (i.e., BF and AG). However, the significance between the different test products was not reached.

Next, treatment effects at different phylogenetic levels (i.e., phylum and family level) were investigated (Table 2). Results indicated that the enhanced Actinobacteria abundance in the PC vessels upon treatment with BF and BF + AG was mainly attributed to increased levels of *Bifidobacteriaceae*. However, in the DC, increased Actinobacteria levels following BF supplementation were mainly attributed to an increased population of *Coriobacteriaceae*. No clear trend was observed for Actinobacteria levels in the DC region upon treatment with BF + AG. The decreased Bacteroidetes population observed in the PC region using BF and BF + AG was mainly the result of a decreased abundance of *Bacteroidaceae*. Nevertheless, the level of bacterial cells belonging to the *Prevotellaceae* family increased for all treatment conditions in the PC region despite the decreased or unchanged phylum levels. The increased Bacteroidetes level observed in the DC region for all treatment conditions was mainly linked to increased levels of *Bacteroidaceae*. For the AG treatment, in particular, increased levels of *Prevotellaceae* were also observed in the DC region. Although Firmicutes levels remained relatively stable in both colonic regions for all treatment conditions, some changes were observed at the family level. *Lachnospiraceae* were significantly stimulated upon treatment with AG in both colonic regions, whereas *Ruminococcaceae* specifically increased in the DC for all test conditions. In addition, BF and BF + AG treatment increased *Eubacteriaceae* levels, while BF + AG supplementation specifically stimulated the *Christensenellaceae* family. Finally, the increased abundance of the Verrucomicrobia phylum in the DC region following BF and BF + AG administration was mainly attributed to increased levels of *Akkermansiaceae*.

#### 3.2.4. Metabolomics

**Figure 3:**
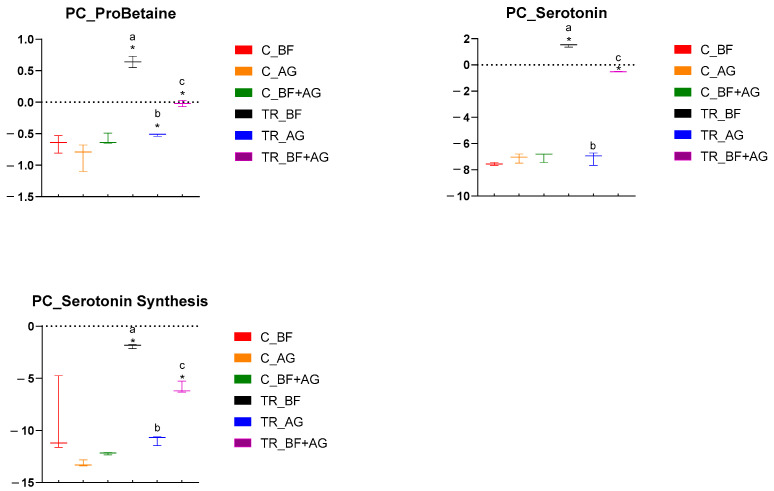
Specific metabolome-related changes observed in the proximal colon (PC) region following long-term product administration. Box plots for different metabolites and metaboINDICATORS (i.e., ProBetaine, serotonin, and serotonin synthesis) detected during the control (C) and treatment (TR) period upon repeated administration of baobab fiber (BF), Arabic gum (AG), and their combination (BF + AG). For each box plot, cleaned, imputed, and log_2_-transformed data from three independent replicates (*n* = 3) were used to determine the minimum, maximum, and median values (log_2_(µM)). Significant differences between corresponding control and treatment values have been indicated by means of ‘*’, while significant differences between the treatments have been indicated by means of a different letter (*p* < 0.05 and q < 0.05).

**Figure 4:**
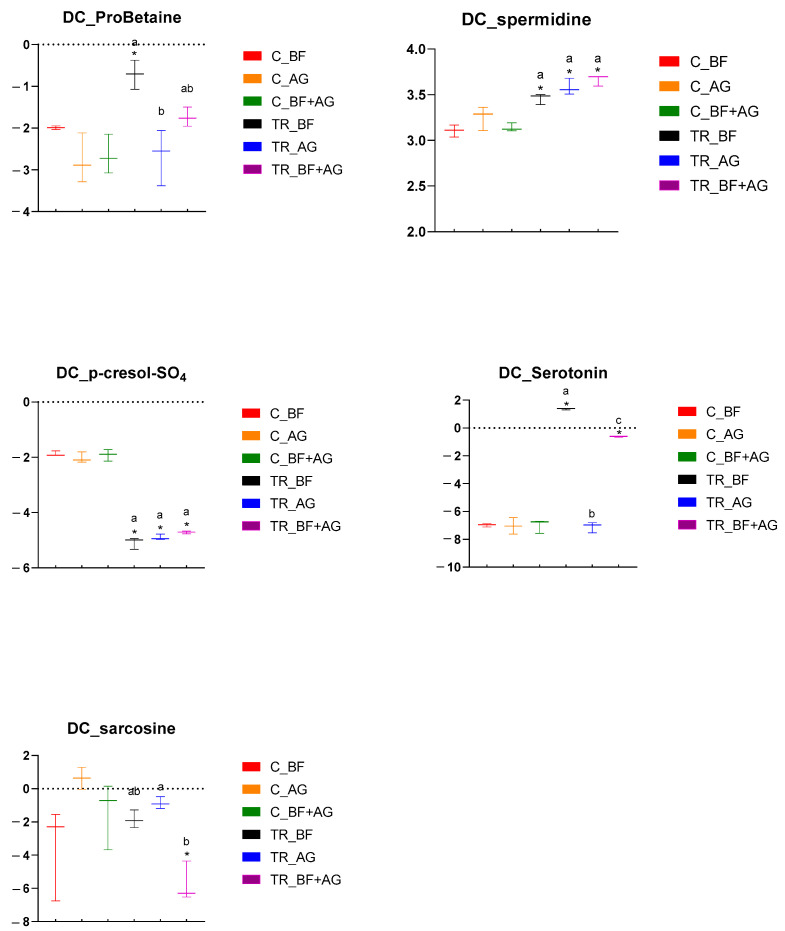
Specific metabolome-related changes observed in the distal colon (DC) region following long-term product administration. Box plots for different metabolites and metaboINDICATORS (i.e., ProBetaine, spermidine, p-cresol-SO_4_, serotonin, and sarcosine) detected during the control (C) and treatment (TR) period upon repeated administration of baobab fiber (BF), Arabic gum (AG), and their combination (BF + AG). For each box plot, cleaned, imputed, and log_2_-transformed data from three independent replicates (*n* = 3) were used to determine the minimum, maximum, and median values (log_2_(µM)). Significant differences between corresponding control and treatment values have been indicated by means of ‘*’, while significant differences between the treatments have been indicated by means of a different letter (*p* < 0.05 and q < 0.05).

Metabolomic analysis was performed on samples collected from the PC and DC reactors of the long-term SHIME^®^ experiment, and these results have been presented by means of separate heatmaps (Figure S2A–F). Six microbiome-related metabolites of interest, detected either in the PC or DC region, were selected and further discussed (Figure 3 and Figure 4). With respect to the PC region (Figure 3), significant changes between the control and treatment period occurred, amongst others, for three metabolites and/or classes/ratios of metabolites, i.e., ProBetaine, serotonin, and serotonin synthesis. For ProBetaine, treatment with BF, AG, and BF + AG resulted in significantly higher concentrations than the corresponding control periods. The highest and lowest values were obtained following treatment with BF and AG, respectively, while intermediate values were obtained for the combined treatment (BF + AG). Treatment with BF and BF + AG resulted in significantly higher serotonin levels as compared to the corresponding control periods, with the strongest effect being observed for the individual BF treatment. In addition, a similar trend was observed for the serotonin synthesis pathway. Treatment with AG alone had no significant effect on the serotonin levels in the PC region, though the serotonin synthesis pathway tended to be boosted. However, significance was only reached for BF and BF + AG. For the DC region (Figure 4), significant changes between the control and treatment period occurred, amongst others, for five metabolites, i.e., ProBetaine, spermidine, p-cresol-SO_4_, serotonin, and sarcosine. For ProBetaine, all treatments increased ProBetaine levels as compared to the corresponding control periods, but significance was only reached upon supplementing BF alone. While the highest and lowest values were obtained for the BF and AG treatment, respectively, intermediate values were obtained for the combined treatment (BF + AG). For spermidine, all treatments significantly augmented concentrations than the corresponding control periods. No significant differences were observed between the treatments, although the combined treatment (BF + AG) proved to result in the highest values. For p-cresol-SO_4_, treatment with BF, AG, and BF + AG significantly reduced the metabolite concentrations compared to the corresponding control periods. As for spermidine, no significant differences were observed between the different test products. Similar to the PC region, treatment with BF and BF + AG resulted in augmented serotonin levels as compared to the control periods, with a more pronounced effect again being observed for the individual BF treatment. Treatment with AG alone had no significant effect on the serotonin levels in the DC region. Finally, the sarcosine concentrations also decreased following treatment with BF, AG, and BF + AG, though only reaching significance for BF + AG. Furthermore, inter-treatment differences were observed, with the combined treatment (BF + AG) resulting in significantly lower sarcosine concentrations compared to AG treatment and intermediate values being obtained following BF supplementation.

#### 3.2.5. Correlation Analysis between Metabolites and Metagenomic Families

**Figure 5:**
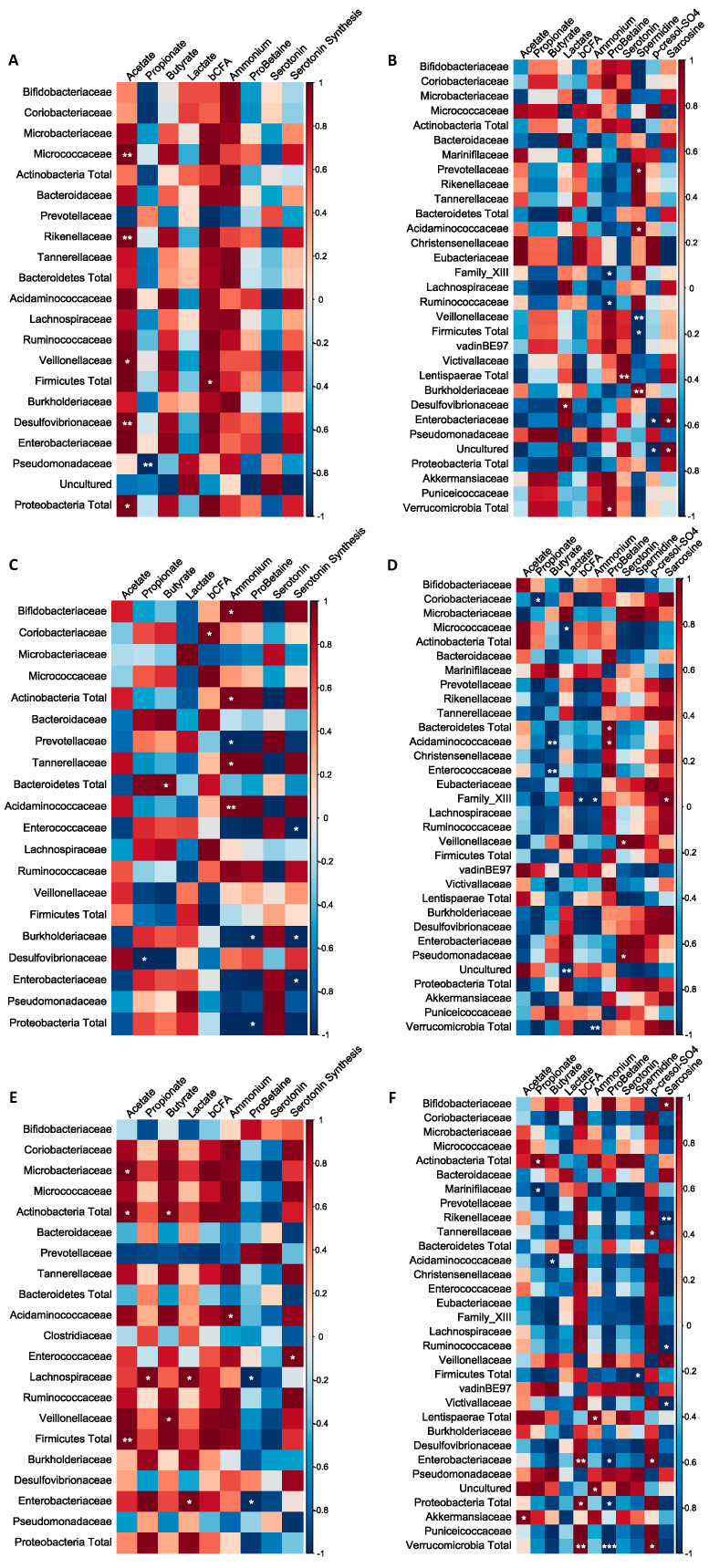
Non-square correlation matrices visualizing pairwise association between the metabolites and metagenomic families or phyla: (**A**) proximal colon–baobab fiber (PC–BF), (**B**) distal colon–baobab fiber (DC–BF), (**C**) proximal colon–Arabic gum (PC–AG), (**D**) distal colon–Arabic gum (DC–AG), (**E**) proximal colon–baobab fiber + Arabic gum (PC–BF + AG), (**F**) distal colon–baobab fiber + Arabic gum (DC–BF + AG). Statistically significant changes are indicated by means of * (*p* ≤ 0.05), ** (*p* ≤ 0.01), or *** (*p* ≤ 0.001).

Correlation analysis was performed in order to identify potential associations between the metabolites of interest and the identified metagenomic taxa (Figure 5). It was overall concluded that the correlations clearly depended on the colonic region (i.e., PC or DC) and the applied treatment (i.e., BF, AG, or BF + AG). In the PC region, a significant positive correlation was, for example, observed between acetate and the *Micrococcaceae*, *Rikenellaceae*, *Veillonellaceae*, and *Desulfovibrionaceae* families upon repeated BF administration. In the DC region, a significant negative correlation was, for example, observed between sarcosine and the *Rikenellaceae*, *Ruminococcaceae*, and *Victivallaceae* families upon repeated BF + AG administration.

## 4. Discussion

### 4.1. The SHIME^®^ Model as a Tool for the In Vitro Simulation of the Gut Microbiota Present in the Different Colon Regions

In the present study, the effect of repeated daily administration of AG, BF, and their combination on the human gut microbiome was investigated using the validated in vitro SHIME^®^ model. While the microbial community of the selected donor was mainly dominated by members of the Actinobacteria, Bacteroidetes, Firmicutes, and Proteobacteria phyla, which are indeed the phyla accounting for 93.5% of all identified species in the human gut microbiome [53], also low abundances of the Lentisphaera, Synergistetes, and Verrucomicrobia phyla were observed. Furthermore, the in vitro microbial community developed in a colon-region-specific way, confirming the literature findings [54]. For example, the *Ruminococcaceae* family (part of *Clostridium* cluster IV) was significantly enhanced in the DC region, as was observed in the study of Van den Abbeele et al. [54]. Furthermore, Van Herreweghen et al. [55] confirmed that the mucin-degrading *Akkermansia muciniphila*, currently the only well-known representative of the Verrucomicrobia phylum in the human gut, specifically colonizes the DC, as was observed within the presented study. Thus, this region-specific colonization indicates that the SHIME^®^ is a highly relevant in vitro model to simulate the effect of repeated administration of BF, AG, and their combination on the metabolic activity and community composition of the human gut microbiota.

### 4.2. Effect of Boabab Fiber and/or Arabic Gum on the Overall Gut Microbial Activity

Administration of BF, AG, and BF + AG enhanced levels of acetate and lactate, which are both key metabolites formed during primary substrate fermentation, indicating an effective breakdown of the test products. While acetate serves as an important energy source for the host [3], increased lactate levels can be considered favorable since it exerts strong antimicrobial effects against pathogens, especially at low pH values [56]. The highest final levels of acetate in the PC and DC region were obtained for BF and AG, respectively, indicating that BF was mainly fermented in the PC region, while AG was also (partially) fermented in the DC region. This region-specific fermentation was most likely the result of a difference in molecule structure/complexity, i.e., the structures of AG and BF were, respectively, reported as being complex [15,17,20] and relatively simple [31]. As previous studies indicated that most chronic colonic diseases mainly originate in the DC [14], prebiotic compounds should preferably exert their biological activity throughout the entire colon, suggesting the potential of co-supplementing BF and AG. Moreover, while the strongest increase in acetate production was obtained following administration of the individual compounds, significantly higher acetate levels were still obtained in both colonic regions upon combined BF + AG treatment, suggesting the potential to lower the test dose without losing the ability to exert prebiotic properties. 

Next to increased levels of acetate and lactate, propionate and butyrate concentrations were also enhanced upon supplementation of the different test products, except for propionate in the DC region upon BF treatment. Butyrate has been linked with anti-inflammatory properties as it is responsible for maintaining intestinal homeostasis [57]. Moreover, it is able to reduce oxidative stress in the colon and plays a protective role against CRC and colitis [58].

Finally, ammonium and bCFA levels decreased for all treatment conditions, an effect that can be considered as beneficial as markers of proteolytic fermentation have been linked with direct and indirect health effects such as cancer development [59]. Although the combined treatment resulted in the highest final bSCFA and ammonium concentrations, probably due to the lower test dose, these levels were generally not significantly different from the individual treatment conditions, confirming that the lower combined treatment dosage was able to still entail potential health-promoting effects.

### 4.3. Effect of Boabab Fiber and/or Arabic Gum on the Gut Microbial Community Composition

The in vivo study of Calame et al. [21] previously confirmed that repeated administration of AG resulted in an increased abundance of *Bifidobacteriaceae* (Actinobacteria phylum)*,* while the current long-term in vitro study is the first one to reveal a similar phenomenon for BF. Moreover, the current study identified potential complementary effects between both prebiotic fibers since BF and AG resulted in enhanced *Bifidobacteriaceae* levels in different colonic regions, i.e., in the PC and DC, respectively. Nevertheless, this complementarity was only confirmed in the PC region where the combined treatment, using a lower dosing strategy as compared to the individual treatments, still resulted in enhanced *Bifidobacteriaceae* levels. Interestingly, the community composition at the operational taxonomic unit (OTU) level (Table S3) indicated that administration of BF and AG resulted in an increased abundance of OTUs related to *Bifidobacterium adolescentis* and *Bifidobacterium longum*, respectively, which again suggests complementarity between both prebiotic compounds. The presence of *Bifidobacteriaceae* in the human gut microbiome can be considered beneficial since previous studies indicated that these bacteria have, amongst others, the potential to (i) prevent and/or treat CRC, (ii) reduce symptoms of inflammatory bowel disease (IBD), and (iii) treat diarrhea [60].

At the level of the Bacteroidetes phylum, all treatments resulted in significantly increased *Bacteroidaceae* and *Prevotellaceae* levels in the DC and PC regions, respectively. For the DC region, in particular, a significantly enhanced *Prevotellaceae* abundance was only observed for AG. Thus, the current study is in line with the study of Kishimoto et al. [61], where a pig model indicated that *Prevotella ruminicola* was the predominant species responsible for AG fermentation and corresponding SCFA production.

In both colonic regions, but for the DC region in particular, increased levels of the *Ruminococcaceae* family (Firmicutes phylum) were observed for all treatments. Interestingly, the community composition results at the OTU level (Table S3) indicated that the administration of AG and BF resulted in an increased abundance of OTU 13 in the PC and DC regions, respectively. This specific OTU number is closely related to *Faecalibacterium prausnitzii*, which is able to exert a strong anti-inflammatory activity in the intestinal environment, mainly linked with the production of butyrate stimulation of regulatory T-cells [62]. Furthermore, increased *Faecalibacterium prausnitzii* levels have been associated with a reduction of endotoxemia in obese subjects [63]. A specific finding for the combinatory product (BF + AG) included the significant stimulation of the *Christensenellaceae* (Firmicutes phylum) abundance in the DC, which was not observed upon supplementation of the individual test compounds. *Christensenellaceae* include several butyrate-producing microorganisms and have been related to many health-promoting effects [64,65]. The study of Goodrich et al. [66], for example, indicated that the abundance of *Christensenellaceae* was significantly increased in individuals with a normal body mass index (BMI) compared to obese individuals, suggesting an anti-obesity effect of this bacterial family. In addition, long-term administration of *Christensenellaceae minuta* DSM33407 in a diet-induced obesity mouse model indicated that this specific strain had a positive effect on body weight gain, food metabolization, and fat accumulation [67].

At the level of the Verrucomicrobia phylum, an enhanced *Akkermansiaceae* abundance was observed for BF alone and the combined treatment (BF + AG). Interestingly, despite the lower test dose being applied, higher *Akkermansiaceae* levels were observed for the combined treatment as compared to BF treatment alone, indicating complementary effects between both fibers. *A. muciniphila*, the most well-known representative of the *Akkermansiaceae* family in the human gut, has anti-inflammatory properties and a positive effect on metabolic health [68]. In mice, *A. muciniphila* was linked with lowering body fat mass, improving glucose homeostasis, and decreasing adipose tissue inflammation [69,70,71]. In overweight and obese persons, on the other hand, a high abundance of *A. muciniphila* was correlated with lower fasting glucose levels, reduced waist-to-hip ratios, and lower subcutaneous adipocyte diameters. In addition, a high *A. muciniphila* abundance at baseline was linked with better glucose homeostasis, blood lipid profile, and body composition by the end of a 6-week calorie restriction period [72]. Thus, the increased *Akkermansiaceae* levels in the DC region indicate a strong prebiotic potential for the combination of BF and AG when supplemented at a concentration of 2.5 g/d.

### 4.4. Effect of Baobab Fiber and/or Arabic Gum on the Metabolomic Profile

For several of the metabolome-related metabolites of interest, similar trends were observed for the individual and combined treatment strategy, including an increase of ProBetaine in the PC, an increase of spermidine in the DC, and a reduction of p-cresol-SO_4_ in the DC. ProBetaine is generally produced by microbial metabolization of betaine, the latter being available through dietary intake and/or synthesis via microbial oxidation of choline [73]. As no betaine was administered during the present in vitro study, the enhanced levels of ProBetaine probably originated from enhanced microbial betaine synthesis, with increased levels of betaine being correlated with antioxidative [73], osmo-protective [74], and anti-inflammatory effects [75]. Spermine and spermidine are essential for the proliferation of eukaryotic cells [76] and have been linked with health-promoting properties, including protection against oxidative stress, maintenance of the intestinal mucosal barrier, and anti-inflammatory effects [77]. In addition, increased spermidine and spermine concentrations were previously linked with a decrease in body weight and an increased glucose tolerance in diet-induced obese mice (models) [78,79]. Thus, the increased microbial production in the DC region can be considered beneficial. P-cresol-SO_4_, which is predominantly synthesized by the gut microbiota in the distal part of the colon, has been linked with detrimental health effects mainly due to oxidative stress [80,81]. Therefore, the reduction of these metabolites in the DC could be considered beneficial.

Also, some product-specific effects were observed at the metabolomic level. Serotonin levels increased in the PC and DC region following long-term administration of BF and BF + AG, while the individual treatment with AG had no direct effect on the serotonin levels. However, the serotonin synthesis pathway tended to be boosted in the PC region only upon administration of AG. Serotonin is a neurotransmitter that is critical for the development and functioning of the central nervous system. Most serotonin (i.e., 95%) is produced in the intestinal environment, where it is involved in hormonal, autocrine, paracrine, and endocrine actions [82]. As an example, serotonin has been found to modulate motility, inflammation, and epithelial development. It is involved in the regulation of peristaltic movement, but high serotonin levels are potentially correlated with increased pathogenesis in IBS patients. In addition, increased mucosal serotonin levels were previously linked with a proinflammatory effect [82]. Therefore, long-term intake of the combined treatment, entailing a more limited increase as compared to BF alone, would be recommended in order to find a balance between the critical functions of serotonin and its adverse side effects when too high levels are being reached. Finally, sarcosine levels decreased in the DC region following long-term administration of the combined treatment strategy, while the individual compounds resulted in unchanged levels. Sarcosine is an intermediate during glycine synthesis with neuroprotective properties [83]. Thus, the production of sarcosine in the colonic environment can be considered beneficial.

### 4.5. Correlation between the Different Metagenomic Taxa and the Metabolites of Interest

As indicated before, significantly enhanced acetate and lactate production was observed in both colonic regions for all treatments. In the literature, enhanced acetate and lactate levels were previously linked with enhanced *Bifidobacterium* spp. levels [84]. However, significant correlations between the *Bifidobacteriaceae* abundance and the production of acetate and lactate were not observed in the current study. Nevertheless, this does not necessarily mean that no acetate and lactate were formed by the members of the *Bifidobacteriaceae* family since acetate and lactate can be converted to secondary metabolites such as butyrate due to cross-feeding interactions within the gut microbial community.

The *Prevotellaceae* abundance was significantly enhanced in the PC region for all treatments. According to the literature, members of this family are able to produce acetate and succinate as a result of their metabolic activity [85]. This was not confirmed in the current study since negative (non-significant) correlations were observed between the *Prevotellaceae* abundance and the production of acetate. As for the *Bifidobacteriaceae* family, this does not necessarily mean that the members of the *Prevotellaceae* family did not contribute to the increased production of acetate, as acetate was likely converted to secondary metabolites such as propionate or butyrate.

Concerning the Firmicutes phylum, an enhanced *Ruminococcaceae* abundance was observed in the DC region for all treatments, while the *Christensenellaceae* abundance in the DC region was only enhanced for the combined treatment. Members of the *Ruminococcaceae* family are able to produce acetate, lactate, succinate, and butyrate, with *Faecalibacterium prausnitzii* being an important butyrate producer [86]. The *Christensenellaceae* members mainly produce acetate, but small amounts of butyrate can also be formed [64]. The *Ruminococcaceae* abundance was indeed positively correlated with acetate and/or lactate production, but significance was not reached. For the *Christensenellaceae* abundance, on the other, a positive correlation with acetate production was indeed observed upon administration of BF + AG, but significance was again not reached.

Members of the *Akkermansiaceae* family were previously linked with an increased acetate and propionate production, with propionate being the most important endpoint [87]. Treatment with BF and BF + AG resulted in an enhanced *Akkermansiaceae* abundance in the DC region, which was indeed positively related with an enhanced acetate and propionate production. However, significance was only reached for the acetate production upon repeated administration of BF + AG.

The study of Gryp et al. [88] observed that the generation of p-cresol was correlated with bacterial species belonging to the *Bacteroidaceae*, *Bifidobacteriaceae*, *Eubacteriaceae*, *Lachnospiraceae*, *Porphyromonadaceae*, *Ruminococcaceae*, and *Veillonellaceae*. While positive correlations between the p-cresol-SO_4_ levels and the abundance of *Eubacteriaceae* (all treatments), *Lachnospiraceae* (AG and BF + AG), *Ruminococcaceae* (AG and BF + AG), and *Veillonellaceae* (AG) were indeed observed within currently presented studies, significance was not reached.

Finally, previous correlation studies have shown that high sarcosine levels were positively correlated with *Escherichia-Shigella* and negatively correlated with *Ruminococcaceae*, *Lachnospiraceae*, *Enterorhabdus*, and *Bacteroides* [89]. Therefore, the reduction in sarcosine concentration after co-supplementation of AG and BF could indicate a shift in microbiome composition away from *Escherichia-Shigella*, a group containing several pathogenic species, towards the other mentioned bacterial taxa, with the latter being confirmed in the current study (i.e., a negative correlation with the *Ruminococcaceae* and *Lachnospiraceae* family was observed, though only reaching significance for the *Ruminococcaceae* family).

## 5. Conclusions

This in vitro research is the first highlighting prebiotic activities of baobab fiber and indicated that repeated administration of baobab fiber, Arabic gum, and their combination had a significant effect on the metabolic activity and the microbial community composition of the gut microbiota present in the proximal and distal colon. Main prebiotic activities have been confirmed throughout lactate, short-chain fatty acid, diversity index, and *Bifidobacterium* enrichment. Despite the lower dosage strategy, co-supplementation of baobab fiber and Arabic gum resulted in some specific synergistic prebiotic effects, including the ability to exert their biological activity throughout the entire colon, specifically increased abundance of *Akkermansiaceae* and *Christensenellaceae* in the distal colon region, and increased levels of spermidine in the DC region.

## Figures and Tables

**Table 1:** Microbial metabolic activity during the long-term SHIME^®^ experiment. Absolute values (average ± stdev) of acetate (mM), propionate (mM), butyrate (mM), lactate (mM), bCFA (mM), and ammonium (mg/L) associated with the baobab fiber (BF), Arabic gum (AG), and combined (BF + AG) treatment in the proximal colon (PC) and distal colon (DC) vessels. Samples were taken during two control (C) and three treatment (TR) weeks (three samples/week). Statistically significant differences between the control and treatment periods are indicated in bold (*p* < 0.05).

	BF				AG				BF + AG			
	PC		DC		PC		DC		PC		DC	
	C	T	C	T	C	T	C	T	C	T	C	T
Acetate (mM)	30.4 ± 1.6	**61.0 ± 8.8**	41.0 ± 2.1	**74.0 ± 11.8**	25.7 ± 2.1	**57.9 ± 14.8**	42.6 ± 2.4	**80.7 ± 15.8**	28.7 ± 1.0	**41.3 ± 4.7**	42.6 ± 0.9	**59.5 ± 6.8**
Propionate (mM)	28.1 ± 2.3	**32.3 ± 3.4**	29.0 ± 2.3	29.4 ± 2.1	23.3 ± 1.7	**33.0 ± 4.0**	24.9 ± 1.8	**33.7 ± 3.7**	26.0 ± 1.7	**32.0 ± 2.2**	25.7 ± 0.9	**31.4 ± 2.8**
Butyrate (mM)	14.6 ± 2.1	**26.8 ± 4.8**	15.3 ± 1.3	**21.0 ± 3.6**	15.1 ± 1.5	**25.4 ± 5.9**	15.5 ± 1.3	**21.3 ± 3.6**	13.4 ± 1.1	**19.3 ± 3.6**	13.8 ± 0.6	**16.4 ± 2.6**
Lactate (mM)	0.015 ± 0.005	**0.035 ± 0.004**	0.008 ± 0.004	**0.026 ± 0.007**	0.017 ± 0.002	**0.027 ± 0.004**	0.009 ± 0.001	**0.017 ± 0.003**	0.018 ± 0.003	**0.025 ± 0.006**	0.008 ± 0.004	**0.018 ± 0.007**
bCFA (mM)	**2.49 ± 0.14**	1.57 ± 0.18	**2.93 ± 0.13**	1.84 ± 0.32	**2.40 ± 0.15**	1.79 ± 0.19	**2.88 ± 0.11**	1.89 ± 0.30	**2.38 ± 0.16**	1.89 ± 0.18	**2.83 ± 0.09**	2.08 ± 0.26
Ammonium (mg/L)	**284 ± 25**	166 ± 39	**402 ± 21**	228 ± 63	**266 ± 28**	189 ± 41	**351 ± 58**	210 ± 57	**265 ± 31**	192 ± 28	**347 ± 49**	247 ± 46

**Table 2:** Treatment effect on the microbial community composition at phylum and family level during the long-term SHIME^®^ experiment. Relative levels (log (cells/mL)) of different families belonging to specific phyla observed in the proximal colon (PC) and distal colon (DC) vessels upon treatment with baobab fiber (BF), Arabic gum (AG), and the combination of both (BF + AG). Relative values were obtained by subtracting the average levels obtained at the end of the control period (*n* = 3) from the corresponding levels obtained at the end of the treatment period (*n* = 3). The intensity of the shading correlates with the relative abundance normalized for each of the different families (i.e., within each row), with the highest intensity being correlated with the highest value. Statistically significant differences between the absolute levels at the end of the control period and the absolute levels at the end of the treatment period were indicated by means of ‘*’ (*p* < 0.05).

Phylum	Family	PC			DC		
		BF	AG	BF + AG	BF	AG	BF + AG
Actinobacteria	*Bifidobacteriaceae*	0.51 *	0.29	0.55 *	0.31	0.20 *	0.00
	*Coriobacteriaceae*	0.44	−0.59 *	−0.73 *	0.55 *	−0.56 *	−0.46 *
	*Microbacteriaceae*	0.87	−0.92	−0.76	0.49	−0.87	−0.73 *
	*Micrococcaceae*	−0.50	0.23	0.07	0.11	0.14	0.13
	Total	0.48 *	0.23	0.40 *	0.31 *	0.13	−0.07
Bacteroidetes	*Bacteroidaceae*	−0.80 *	−0.08	−0.41 *	0.22 *	0.25 *	0.28 *
	*Marinifilaceae*	<LOQ	<LOQ	<LOQ	0.67	0.39	0.29
	*Prevotellaceae*	1.40 *	2.96 *	1.34 *	0.68	1.59 *	0.75
	*Rikenellaceae*	−0.72 *	<LOQ	<LOQ	0.28	0.11	0.21
	*Tannerellaceae*	−0.98 *	0.44 *	0.10	−0.37 *	−0.23 *	−0.01
	Total	−0.47 *	0.13	−0.34 *	0.20 *	0.25 *	0.27 *
Firmicutes	*Acidaminococcaceae*	−0.88 *	−0.21	−0.45	−0.23	0.08	0.04
	*Christensenellaceae*	<LOQ	<LOQ	<LOQ	0.48	−0.56 *	1.14 *
	*Clostridiaceae_1*	<LOQ	−0.31	−1.03	<LOQ	<LOQ	<LOQ
	*Enterococcaceae*	<LOQ	−0.46	−0.25	<LOQ	−0.56 *	0.65
	*Eubacteriaceae*	<LOQ	<LOQ	<LOQ	0.65 *	0.31	0.45 *
	*Family_XIII*	<LOQ	<LOQ	<LOQ	0.10	−0.55 *	−0.14
	*Lachnospiraceae*	−0.09	0.46 *	0.00	0.12	0.23 *	0.04
	*Ruminococcaceae*	0.82	0.72	0.91	0.85 *	0.71 *	0.67 *
	*Veillonellaceae*	−0.09	−0.18	−0.22 *	−0.39	−0.16 *	−0.03
	Total	−0.08	−0.05	−0.19 *	−0.11	0.03	0.03
Lentisphaerae	*vadinBE97*	<LOQ	<LOQ	<LOQ	−0.16 *	−0.86 *	−0.65 *
	*Victivallaceae*	<LOQ	<LOQ	<LOQ	0.55	−0.99 *	0.10
	Total	<LOQ	<LOQ	<LOQ	0.41	−0.95 *	−0.32 *
Proteobacteria	*Burkholderiaceae*	−0.47 *	0.17	−0.48	−0.04	−0.02	−0.09
	*Desulfovibrionaceae*	−1.48	<LOQ	0.15	−0.17	−0.31	−0.08
	*Enterobacteriaceae*	0.09	−0.74	−0.58	0.28	−0.99	0.42
	*Pseudomonadaceae*	0.19	−0.30	−0.71	0.26	0.17	0.38 *
	*uncultured*	−1.89 *	<LOQ	<LOQ	−0.57	0.87	1.34
	Total	−0.22	−0.54	−0.59	−0.02	−0.18	0.21
Verrucomicrobia	*Akkermansiaceae*	<LOQ	<LOQ	<LOQ	0.95 *	−0.11	1.26 *
	*Puniceicoccaceae*	<LOQ	<LOQ	<LOQ	0.96	<LOQ	−0.11
	Total	<LOQ	<LOQ	<LOQ	0.95 *	−0.33	0.75 *

## Data Availability

The raw data supporting the conclusions of this article will be made available by the authors on request.

## Supplementary Materials

The following supporting information can be downloaded at https://www.mdpi.com/article/10.3390/nu16111570/s1. Figure S1: Donor-specific metabolic activity effect during the short-term fecal batch fermentation experiments. Principle component analysis (PCA) of all metabolic activity results obtained from the short-term fecal batch fermentation experiments (i.e., pH, acetate, propionate, butyrate, bCFA, and total SCFA). Relative changes between 0 and 48 h of incubation were normalized for all donors (i.e., donor A, donor B, and donor C) and all test conditions (i.e., blank, 2.5 or 5 g/L baobab fiber (BF), 2.5 or 5 g/L Arabic gum (AG), and 2.5 or 5 g/L BF + AG). In addition, normalized values were calculated for an ‘average donor’ to identify donor-specific effects. A confidence level of 70% was selected, and the PCA was performed based on three biological replicates (= three donors) and two technical replicates. Figure S2: Metabolomic profile following long-term product administration. Heatmaps of the metabolites and metaboINDICATORS that were significantly different (*p* < 0.05 and q < 0.05) in the relevant group comparisons after data cleaning, imputation, and log_2_-transformation. Separate heatmaps have been included for each combination of the test product (i.e., baobab fiber (BF), Arabic gum (AG), and their combination (BF + AG)) and each colonic region (i.e., proximal colon (PC) and distal colon (DC)). (A) PC–BF, (B) PC–AG, (C) PC–BF + AG, (D) DC–BF, (E) DC–AG, and (F) DC–BF + AG. Each row and column of the heatmap represent a different metabolite and sample, respectively. For each metabolite, it was indicated as well as to which category of metabolites (see Table S1) it belongs. For both the control (C) and treatment (TR) periods, three replicates (*n* = 3) have been included, and the representative log_2_-transformed values have been indicated by means of a specific color range going from red to blue. Table S1: Classification of metabolome-related metabolites. Metabolite categories identified following targeted ultra-high-performance liquid chromatography coupled with high-resolution Orbitrap mass spectrometry (UHPLC-LC-MLS/MS). Table S2: Region-specific microbial colonization in the SHIME^®^. Average (± SD) levels (log (cells/mL)) of different bacterial families encountered in all proximal colon (PC) and distal colon (DC) vessels during the last week of the control period (*n* = 9). The *p*-value has been indicated in bold when significant differences (*p* < 0.05) were obtained between the different colon regions. In addition, the intensity of the shading correlates with the absolute abundance, normalized for each of the different families (i.e., within each row). Table S3: Treatment effect on the microbial community composition at OTU level during the long-term SHIME^®^ experiment. Relative levels (log (cells/mL)) of the 25 most abundant OTUs, belonging to specific phyla and families, observed in the proximal colon (PC) and distal colon (DC) vessels upon treatment with baobab fiber (BF), Arabic gum (AG), and the combination of both (BF + AG). The closely related species, as identified by blasting of the corresponding sequence, were also included for each OTU. Relative values were obtained by subtracting the average levels obtained at the end of the control period (*n* = 3) from the corresponding levels obtained at the end of the treatment period (*n* = 3). The intensity of the shading correlates with the relative abundance, normalized for each of the different OTUs (i.e., within each row). Statistically significant differences between the absolute levels at the end of the control period and the absolute levels at the end of the treatment period were indicated by means of ‘*’ (*p* < 0.05).
