# Supplementary material for: Co-Supplementation of Baobab Fiber and Arabic Gum Synergistically Modulates the In Vitro Human Gut Microbiome Revealing Complementary and Promising Prebiotic Properties
Source: Nutrients. 2024 May 22;16(11):1570. doi: 10.3390/nu16111570 (PMC11173755; doi:10.3390/nu16111570)
Supplement: Supplementary file 1 [file nutrients-16-01570-s001.zip › Supplementary Figure S1.pdf]

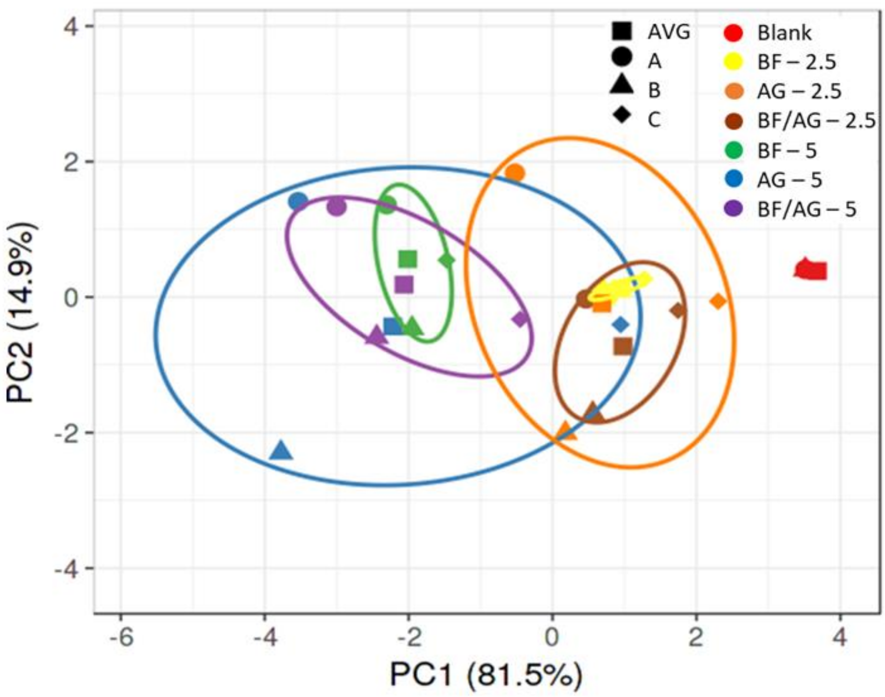

Figure S1. Donor-specific metabolic activity effect during the short-term fecal batch fermentation experiments. Principle component analysis (PCA) of all metabolic activity results obtained from the short-term fecal batch fermentation experiments (i.e., pH, acetate, propionate, butyrate, bCFA, and total SCFA). Relative changes between 0 and 48 h of incubation were normalized for all donors (i.e., donor A, donor B, and donor C) and all test conditions (i.e., blank, 2.5 or 5 g/L baobab fiber (BF), 2.5 or 5 g/L arabic gum (AG), and 2.5 or 5 g/L BF + AG). In addition, normalized values were calculated for an 'average donor' to identify donor-specific effects. A confidence level of 70% was selected and the PCA was performed based on three biological replicates (= three donors) and two technical replicates.
