# Supplementary material for: Co-Supplementation of Baobab Fiber and Arabic Gum Synergistically Modulates the In Vitro Human Gut Microbiome Revealing Complementary and Promising Prebiotic Properties
Source: Nutrients. 2024 May 22;16(11):1570. doi: 10.3390/nu16111570 (PMC11173755; doi:10.3390/nu16111570)
Supplement: Supplementary file 1 [file nutrients-16-01570-s001.zip › Supplementary Figure S2.pdf]

A

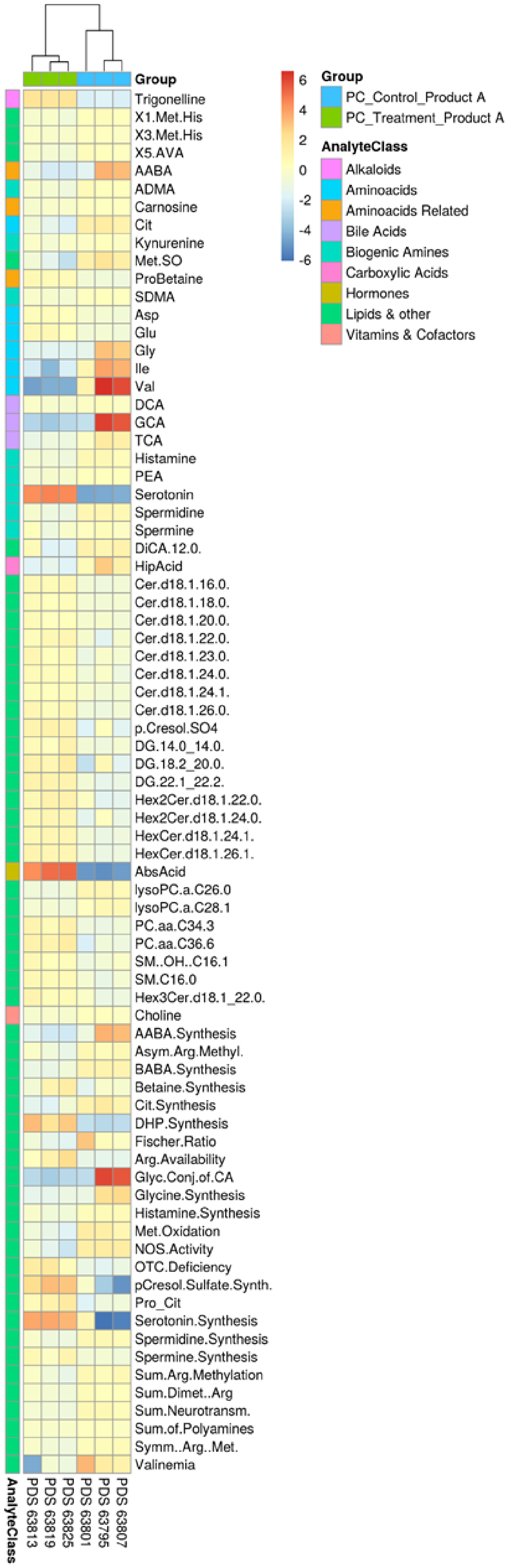
Group
Trigonelline
X1.Met.His
X3.Met.His
X5.AVA
AABA
ADMA
Carnosine
Cit
Kynurenine
Met.SO
ProBetaine
SDMA
Asp
Glu
Gly
Ile
Val
DCA
GCA
TCA
Histamine
PEA
Serotonin
Spermidine
Spermine
DiCA.12.0.
HipAcid
Cer.d18.1.16.0.
Cer.d18.1.18.0.
Cer.d18.1.20.0.
Cer.d18.1.22.0.
Cer.d18.1.23.0.
Cer.d18.1.24.0.
Cer.d18.1.24.1.
Cer.d18.1.26.0.
p.Cresol.SO4
DG.14.0_14.0.
DG.18.2_20.0.
DG.22.1_22.2.
Hex2Cer.d18.1.22.0.
Hex2Cer.d18.1.24.0.
HexCer.d18.1.24.1.
HexCer.d18.1.26.1.
AbsAcid
lysoPC.a.C26.0
lysoPC.a.C28.1
PC.aa.C34.3
PC.aa.C36.6
SM..OH..C16.1
SM.C16.0
Hex3Cer.d18.1_22.0.
Choline
AABA.Synthesis
Asym.Arg.Methyl.
BABA.Synthesis
Betaine.Synthesis
Cit.Synthesis
DHP.Synthesis
Fischer.Ratio
Arg.Availability
Glyc.Conj.of.CA
Glycine.Synthesis
Histamine.Synthesis
Met.Oxidation
NOS.Activity
OTC.Deficiency
pCresol.Sulfate.Synth.
Pro_Cit
Serotonin.Synthesis
Spermidine.Synthesis
Spermine.Synthesis
Sum.Arg.Methylation
Sum.Dimet..Arg
Sum.Neurotransm.
Sum.of.Polyamines
Symm..Arg..Met.
Valinemia
AnalyteClass
PDS 63813
PDS 63819
PDS 63825
PDS 63801
PDS 63795
PDS 63807
6
4
2
0
-2
-4
-6
Group
PC_Control_Product A
PC_Treatment_Product A
AnalyteClass
Alkaloids
Aminoacids
Aminoacids Related
Bile Acids
Biogenic Amines
Carboxylic Acids
Hormones
Lipids & other
Vitamins & Cofactors

B

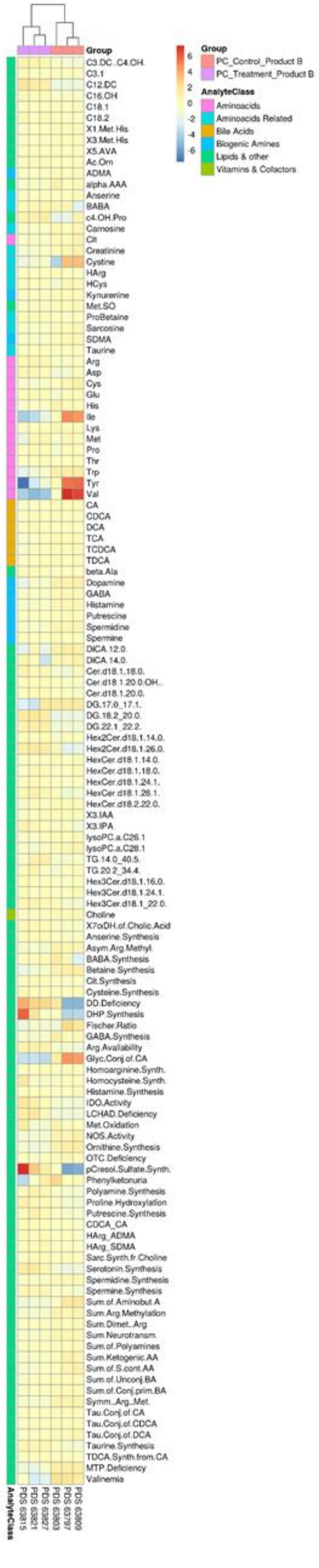
Group
C3.DC..C4.OH.
C3.1
C12.DC
C16.OH
C18.1
C18.2
X1.Met.His
X3.Met.His
X5.AVA
Ac.Orn
ADMA
alpha.AAA
Anserine
BABA
c4.OH.Pro
Carnosine
Cit
Creatinine
Cystine
HArg
HCys
Kynurenine
Met.SO
ProBetaine
Sarcosine
SDMA
Taurine
Arg
Asp
Cys
Glu
His
Ile
Lys
Met
Pro
Thr
Trp
Tyr
Val
CA
CDCA
DCA
TCA
TCDCA
TDCA
beta.Ala
Dopamine
GABA
Histamine
Putrescine
Spermidine
Spermine
DICA.12.0.
DICA.14.0.
Cer.d18.1.18.0.
Cer.d18.1.20.0.OH..
Cer.d18.1.20.0.
DG.17.0_17.1.
DG.18.2_20.0.
DG.22.1_22.2.
Hex2Cer.d18.1.14.0.
Hex2Cer.d18.1.26.0.
HexCer.d18.1.14.0.
HexCer.d18.1.18.0.
HexCer.d18.1.24.1.
HexCer.d18.1.26.1.
HexCer.d18.2.22.0.
X3.IAA
X3.IPA
lysoPC.a.C26.1
lysoPC.a.C28.1
TG.14.0_40.5.
TG.20.2_34.4.
Hex3Cer.d18.1.16.0.
Hex3Cer.d18.1.24.1.
Hex3Cer.d18.1_22.0.
Choline
X7αDH.of.Cholic.Acid
Anserine.Synthesis
Asym.Arg.Methyl.
BABA.Synthesis
Betaine.Synthesis
Cit.Synthesis
Cysteine.Synthesis
DD.Deficiency
DHP.Synthesis
Fischer.Ratio
GABA.Synthesis
Arg.Availability
Glyc.Conj.of.CA
Homoarginine.Synth.
Homocysteine.Synth.
Histamine.Synthesis
IDO.Activity
LCHAD.Deficiency
Met.Oxidation
NOS.Activity
Ornithine.Synthesis
OTC.Deficiency
pCresol.Sulfate.Synth.
Phenylketonuria
Polyamine.Synthesis
Proline.Hydroxylation
Putrescine.Synthesis
CDCA_CA
HArg_ADMA
HArg_SDMA
Sarc.Synth.fr.Choline
Serotonin.Synthesis
Spermidine.Synthesis
Spermine.Synthesis
Sum.of.Aminobut.A
Sum.Arg.Methylation
Sum.Dimet..Arg
Sum.Neurotransm.
Sum.of.Polyamines
Sum.Ketogenic.AA
Sum.of.S.cont.AA
Sum.of.Unconj.BA
Sum.of.Conj.prim.BA
Symm..Arg..Met.
Tau.Conj.of.CA
Tau.Conj.of.CDCA
Tau.Conj.of.DCA
Taurine.Synthesis
TDCA.Synth.from.CA
MTP.Deficiency
Valinemia
AnalyteClass
PDS 63815
PDS 63821
PDS 63827
PDS 63803
PDS 63797
PDS 63809
6
4
2
0
-2
-4
-6
Group
PC_Control_Product B
PC_Treatment_Product B
AnalyteClass
Aminoacids
Aminoacids Related
Bile Acids
Biogenic Amines
Lipids & other
Vitamins & Cofactors

C

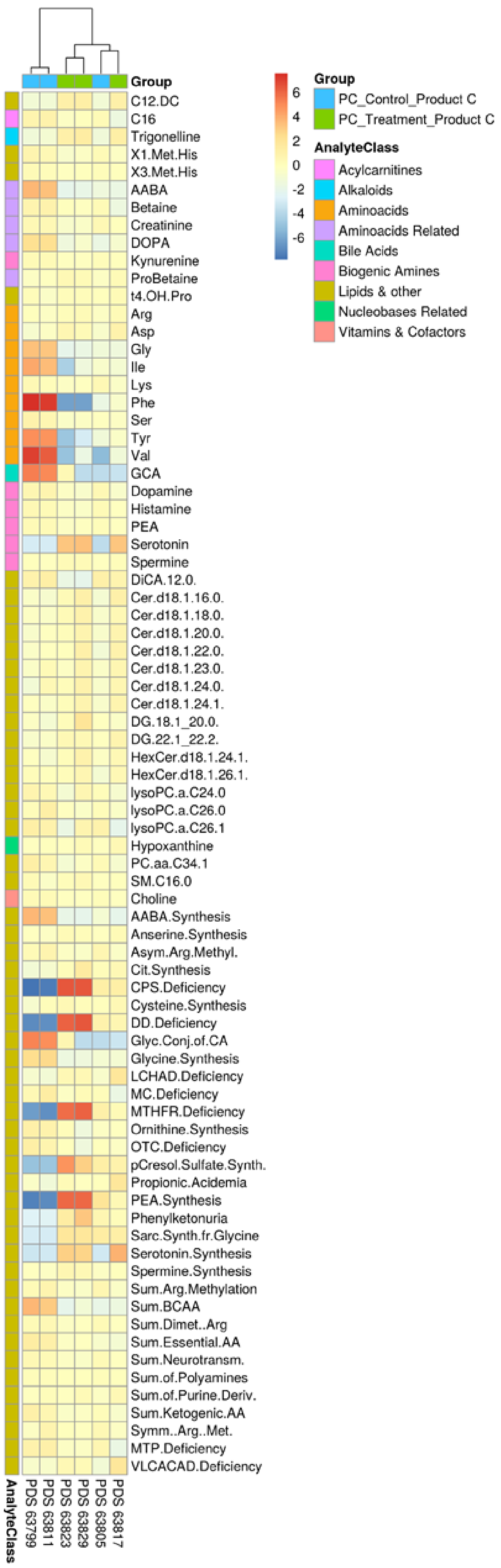

Group
PC_Control_Product C
PC_Treatment_Product C
AnalyteClass
Acylcarnitines
Alkaloids
Aminoacids
Aminoacids Related
Bile Acids
Biogenic Amines
Lipids & other
Nucleobases Related
Vitamins & Cofactors
6
4
2
0
-2
-4
-6
C12.DC
C16
Trigonelline
X1.Met.His
X3.Met.His
AABA
Betaine
Creatinine
DOPA
Kynurenine
ProBetaine
t4.OH.Pro
Arg
Asp
Gly
Ile
Lys
Phe
Ser
Tyr
Val
GCA
Dopamine
Histamine
PEA
Serotonin
Spermine
DiCA.12.0.
Cer.d18.1.16.0.
Cer.d18.1.18.0.
Cer.d18.1.20.0.
Cer.d18.1.22.0.
Cer.d18.1.23.0.
Cer.d18.1.24.0.
Cer.d18.1.24.1.
DG.18.1_20.0.
DG.22.1_22.2.
HexCer.d18.1.24.1.
HexCer.d18.1.26.1.
lysoPC.a.C24.0
lysoPC.a.C26.0
lysoPC.a.C26.1
Hypoxanthine
PC.aa.C34.1
SM.C16.0
Choline
AABA.Synthesis
Anserine.Synthesis
Asym.Arg.Methyl.
Cit.Synthesis
CPS.Deficiency
Cysteine.Synthesis
DD.Deficiency
Glyc.Conj.of.CA
Glycine.Synthesis
LCHAD.Deficiency
MC.Deficiency
MTHFR.Deficiency
Ornithine.Synthesis
OTC.Deficiency
pCresol.Sulfate.Synth.
Propionic.Acidemia
PEA.Synthesis
Phenylketonuria
Sarc.Synth.fr.Glycine
Serotonin.Synthesis
Spermine.Synthesis
Sum.Arg.Methylation
Sum.BCAA
Sum.Dimet..Arg
Sum.Essential.AA
Sum.Neurotransm.
Sum.of.Polyamines
Sum.of.Purine.Deriv.
Sum.Ketogenic.AA
Symm..Arg..Met.
MTP.Deficiency
VLCACAD.Deficiency
AnalyteClass
PDS 63799
PDS 63811
PDS 63823
PDS 63829
PDS 63805
PDS 63817

D

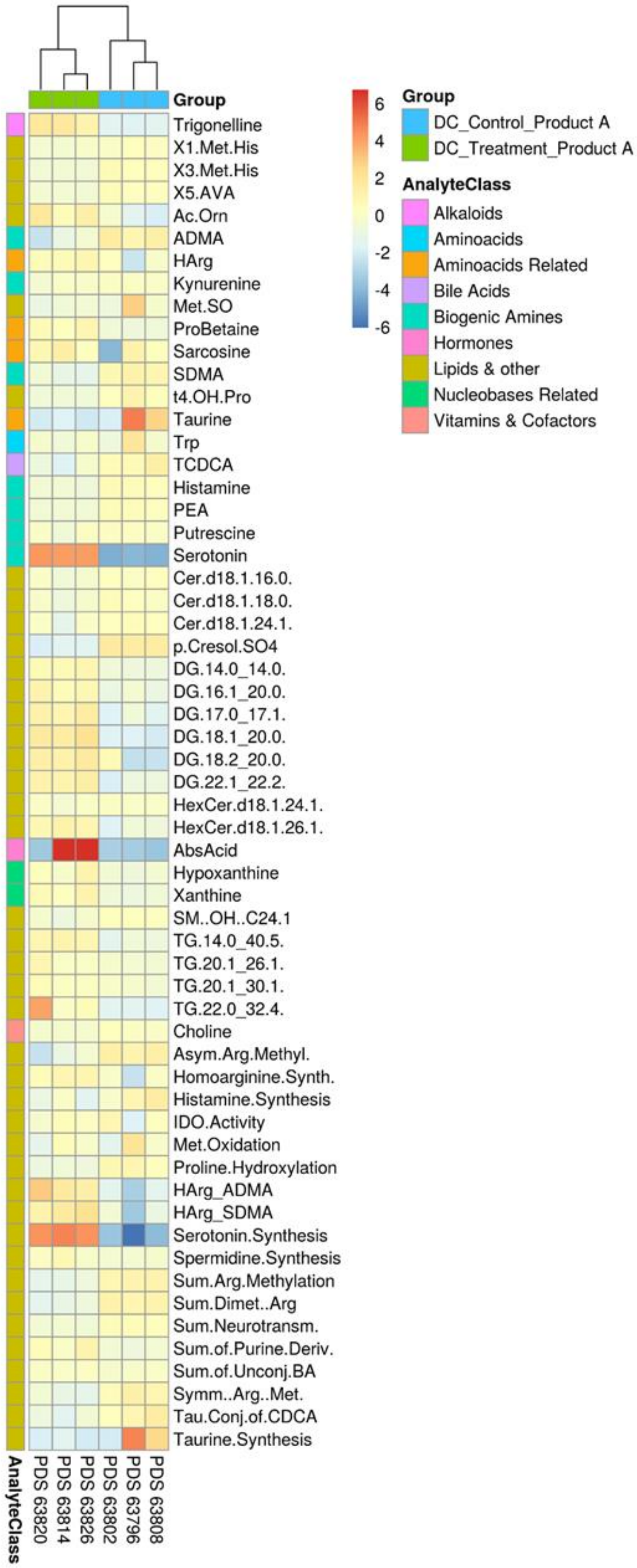

Group
Trigonelline
X1.Met.His
X3.Met.His
X5.AVA
Ac.Orn
ADMA
HArg
Kynurenine
Met.SO
ProBetaine
Sarcosine
SDMA
t4.OH.Pro
Taurine
Trp
TCDCA
Histamine
PEA
Putrescine
Serotonin
Cer.d18.1.16.0.
Cer.d18.1.18.0.
Cer.d18.1.24.1.
p.Cresol.SO4
DG.14.0_14.0.
DG.16.1_20.0.
DG.17.0_17.1.
DG.18.1_20.0.
DG.18.2_20.0.
DG.22.1_22.2.
HexCer.d18.1.24.1.
HexCer.d18.1.26.1.
AbsAcid
Hypoxanthine
Xanthine
SM..OH..C24.1
TG.14.0_40.5.
TG.20.1_26.1.
TG.20.1_30.1.
TG.22.0_32.4.
Choline
Asym.Arg.Methyl.
Homoarginine.Synth.
Histamine.Synthesis
IDO.Activity
Met.Oxidation
Proline.Hydroxylation
HArg_ADMA
HArg_SDMA
Serotonin.Synthesis
Spermidine.Synthesis
Sum.Arg.Methylation
Sum.Dimet..Arg
Sum.Neurotransm.
Sum.of.Purine.Deriv.
Sum.of.Unconj.BA
Symm..Arg..Met.
Tau.Conj.of.CDCA
Taurine.Synthesis
AnalyteClass
PDS 63820
PDS 63814
PDS 63826
PDS 63802
PDS 63796
PDS 63808
6
4
2
0
-2
-4
-6
Group
DC_Control_Product A
DC_Treatment_Product A
AnalyteClass
Alkaloids
Aminoacids
Aminoacids Related
Bile Acids
Biogenic Amines
Hormones
Lipids & other
Nucleobases Related
Vitamins & Cofactors

E

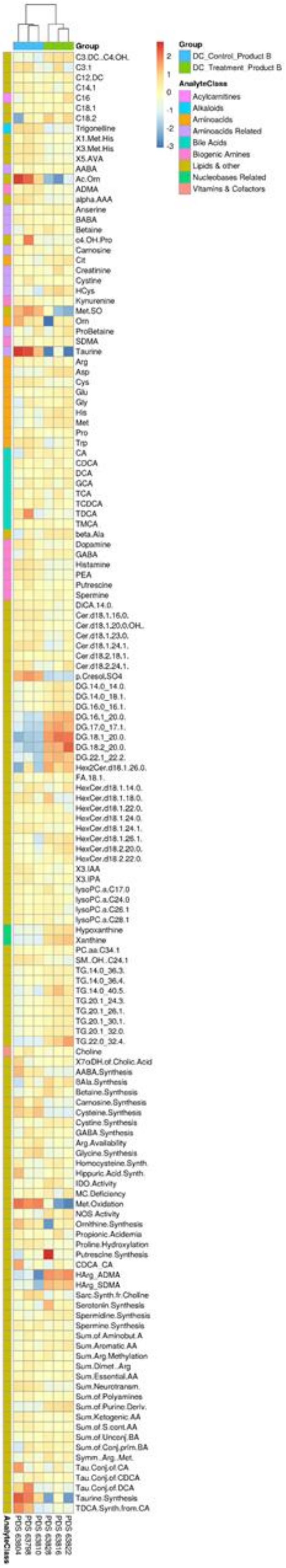
Group
DC_Control_Product B
DC_Treatment_Product B
AnalyteClass
Acylcarnitines
Alkaloids
Aminoacids
Aminoacids Related
Bile Acids
Biogenic Amines
Lipids & other
Nucleobases Related
Vitamins & Cofactors
2
1
0
-1
-2
-3
C3.DC..C4.OH.
C3.1
C12.DC
C14.1
C16
C18.1
C18.2
Trigonelline
X1.Met.His
X3.Met.His
X5.AVA
AABA
Ac.Orn
ADMA
alpha.AAA
Anserine
BABA
Betaine
c4.OH.Pro
Carnosine
Cit
Creatinine
Cystine
HCys
Kynurenine
Met.SO
Orn
ProBetaine
SDMA
Taurine
Arg
Asp
Cys
Glu
Gly
His
Met
Pro
Trp
CA
CDCA
DCA
GCA
TCA
TCDCA
TDCA
TMCA
beta.Ala
Dopamine
GABA
Histamine
PEA
Putrescine
Spermine
DiCA.14.0.
Cer.d18.1.16.0.
Cer.d18.1.20.0.OH.
Cer.d18.1.23.0.
Cer.d18.1.24.1.
Cer.d18.2.18.1.
Cer.d18.2.24.1.
p.Cresol.SO4
DG.14.0_14.0.
DG.14.0_18.1.
DG.16.0_16.1.
DG.16.1_20.0.
DG.17.0_17.1.
DG.18.1_20.0.
DG.18.2_20.0.
DG.22.1_22.2.
Hex2Cer.d18.1.26.0.
FA.18.1.
HexCer.d18.1.14.0.
HexCer.d18.1.18.0.
HexCer.d18.1.22.0.
HexCer.d18.1.24.0.
HexCer.d18.1.24.1.
HexCer.d18.1.26.1.
HexCer.d18.2.20.0.
HexCer.d18.2.22.0.
X3.IAA
X3.IPA
lysoPC.a.C17.0
lysoPC.a.C24.0
lysoPC.a.C26.1
lysoPC.a.C28.1
Hypoxanthine
Xanthine
PC.aa.C34.1
SM..OH..C24.1
TG.14.0_36.3.
TG.14.0_36.4.
TG.14.0_40.5.
TG.20.1_24.3.
TG.20.1_26.1.
TG.20.1_30.1.
TG.20.1_32.0.
TG.22.0_32.4.
Choline
X7αDH.of.Cholic.Acid
AABA.Synthesis
BAla.Synthesis
Betaine.Synthesis
Carnosine.Synthesis
Cysteine.Synthesis
Cystine.Synthesis
GABA.Synthesis
Arg.Availability
Glycine.Synthesis
Homocysteine.Synth.
Hippuric.Acid.Synth.
IDO.Activity
MC.Deficiency
Met.Oxidation
NOS.Activity
Ornithine.Synthesis
Propionic.Acidemia
Proline.Hydroxylation
Putrescine.Synthesis
CDCA_CA
HArg_ADMA
HArg_SDMA
Sarc.Synth.fr.Choline
Serotonin.Synthesis
Spermidine.Synthesis
Spermine.Synthesis
Sum.of.Aminobut.A
Sum.Aromatic.AA
Sum.Arg.Methylation
Sum.Dimet..Arg
Sum.Essential.AA
Sum.Neurotransm.
Sum.of.Polyamines
Sum.of.Purine.Deriv.
Sum.Ketogenic.AA
Sum.of.S.cont.AA
Sum.of.Unconj.BA
Sum.of.Conj.prim.BA
Symm..Arg..Met.
Tau.Conj.of.CA
Tau.Conj.of.CDCA
Tau.Conj.of.DCA
Taurine.Synthesis
TDCA.Synth.from.CA
AnalyteClass
PDS.63804
PDS.63798
PDS.63810
PDS.63828
PDS.63816
PDS.63822

F

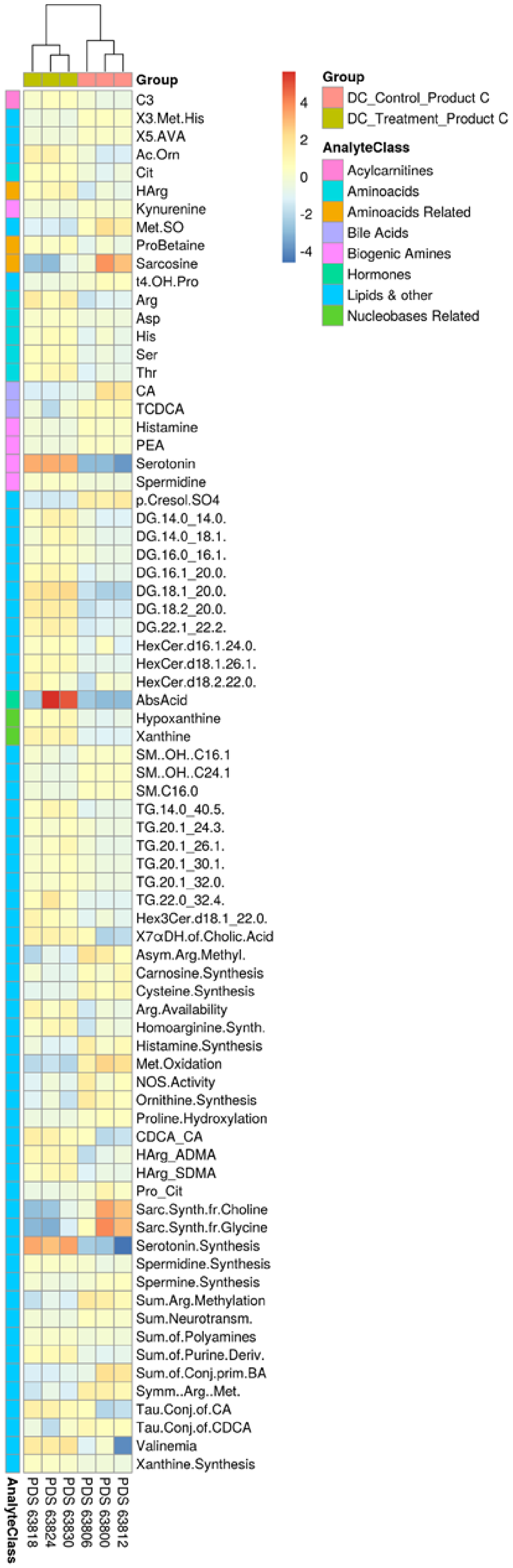

Group
DC_Control_Product C
DC_Treatment_Product C
AnalyteClass
Acylcarnitines
Aminoacids
Aminoacids Related
Bile Acids
Biogenic Amines
Hormones
Lipids & other
Nucleobases Related
4
2
0
-2
-4
C3
X3.Met.His
X5.AVA
Ac.Orn
Cit
HArg
Kynurenine
Met.SO
ProBetaine
Sarcosine
t4.OH.Pro
Arg
Asp
His
Ser
Thr
CA
TCDCA
Histamine
PEA
Serotonin
Spermidine
p.Cresol.SO4
DG.14.0_14.0.
DG.14.0_18.1.
DG.16.0_16.1.
DG.16.1_20.0.
DG.18.1_20.0.
DG.18.2_20.0.
DG.22.1_22.2.
HexCer.d16.1.24.0.
HexCer.d18.1.26.1.
HexCer.d18.2.22.0.
AbsAcid
Hypoxanthine
Xanthine
SM..OH..C16.1
SM..OH..C24.1
SM.C16.0
TG.14.0_40.5.
TG.20.1_24.3.
TG.20.1_26.1.
TG.20.1_30.1.
TG.20.1_32.0.
TG.22.0_32.4.
Hex3Cer.d18.1_22.0.
X7αDH.of.Cholic.Acid
Asym.Arg.Methyl.
Carnosine.Synthesis
Cysteine.Synthesis
Arg.Availability
Homoarginine.Synth.
Histamine.Synthesis
Met.Oxidation
NOS.Activity
Ornithine.Synthesis
Proline.Hydroxylation
CDCA_CA
HArg_ADMA
HArg_SDMA
Pro_Cit
Sarc.Synth.fr.Choline
Sarc.Synth.fr.Glycine
Serotonin.Synthesis
Spermidine.Synthesis
Spermine.Synthesis
Sum.Arg.Methylation
Sum.Neurotransm.
Sum.of.Polyamines
Sum.of.Purine.Deriv.
Sum.of.Conj.prim.BA
Symm..Arg..Met.
Tau.Conj.of.CA
Tau.Conj.of.CDCA
Valinemia
Xanthine.Synthesis
AnalyteClass
PDS 63818
PDS 63824
PDS 63830
PDS 63806
PDS 63800
PDS 63812

Figure S2. Metabolomic profile following long-term product administration. Heatmaps of the metabolites and metaboINDICATORS that were significantly different ($p<0.05$ and $q<0.05$) in the relevant group comparisons after data cleaning, imputation, and log_2-transformation. Separate heatmaps have been included for each combination of test product (i.e., baobab fiber (BF), arabic gum (AG), and their combination (BF + AG)) and each colonic region (i.e., proximal colon (PC) and distal colon (DC)). (A) PC - BF, (B) PC - AG, (C) PC - BF+AG, (D) DC - BF, (E) DC - AG, and (F) DC - BF+AG. Each row and column of the heatmap represent a different metabolite and sample, respectively. For each metabolite, it was indicated as well to which category of metabolites (see Table S1) it belongs. For both the control (C) and treatment (TR) period, three replicates (n=3) have been included and the representative log_2-transformed values have been indicated by means of a specific color range going from red to blue.
