# Supplementary material for: Co-Supplementation of Baobab Fiber and Arabic Gum Synergistically Modulates the In Vitro Human Gut Microbiome Revealing Complementary and Promising Prebiotic Properties
Source: Nutrients. 2024 May 22;16(11):1570. doi: 10.3390/nu16111570 (PMC11173755; doi:10.3390/nu16111570)
Supplement: Supplementary file 1 [file nutrients-16-01570-s001.zip › Supplementary Table S1.pdf]

Table S1. Classification of metabolome-related metabolites. Metabolite categories identified following targeted ultra-high-performance liquid chromatography coupled to high resolution Orbitrap mass spectrometry (UHPLC-LC-MLS/MS).

**Metabolite categories**	
**Small molecule category**	**Lipid category**
Alkaloids	Acylcarnitines
Amino acids	Phosphatidylcholines
Amino acid related	Lysophosphatidylcholines
Bile acids	Sphingomyelins
Biogenic amines	Cholesteryl esters
Carboxylic acids	Ceramides
Cresols	Dihydroceramides
Hormones	Glucosylceramides
Indoles and derivatives	Lactosylceramides
Nucleobases and related	Trihexosylceramides
Vitamins and cofactors	Diacylglycerols
Sugars	Triacylglycerols
