# Supplementary material for: Co-Supplementation of Baobab Fiber and Arabic Gum Synergistically Modulates the In Vitro Human Gut Microbiome Revealing Complementary and Promising Prebiotic Properties
Source: Nutrients. 2024 May 22;16(11):1570. doi: 10.3390/nu16111570 (PMC11173755; doi:10.3390/nu16111570)
Supplement: Supplementary file 1 [file nutrients-16-01570-s001.zip › Supplementary Table S2.pdf]

Table S2. Region-specific microbial colonization in the SHIME®. Average (± SD) levels (log (cells/mL)) of different bacterial families encountered in all proximal colon (PC) and distal colon (DC) vessels during the last week of the control period (n=9). The p-value has been indicated in bold when significant differences (p<0.05) were obtained between the different colon regions. In addition, the intensity of the shading correlates with the absolute abundance, normalized for each of the different families (i.e., within each row).

Phylum	Family	Abundance				p-value
		PC		DC		
		Average	SD	Average	SD	
Actinobacteria	*Bifidobacteriaceae*	8.21	0.10	8.43	0.16	**0.0057**
	Coriobacteriaceae	6.66	0.08	6.82	0.20	0.0862
	Microbacteriaceae	7.40	0.84	7.55	0.65	0.3365
	Micrococcaceae	7.06	0.25	7.22	0.15	0.0604
Bacteroidetes	*Bacteroidaceae*	8.59	0.22	8.74	0.10	**0.0319**
	Bacteroidia_unclassified	<LOQ	0.00	6.26	0.13	**0.0000**
	Barnesiellaceae	<LOQ	0.00	5.99	0.11	**0.0000**
	Marinifilaceae	<LOQ	0.00	6.25	0.14	**0.0000**
	Prevotellaceae	6.27	0.74	6.20	0.32	0.4344
	Rikenellaceae	5.47	1.83	6.83	0.10	**0.0042**
	Tannerellaceae	6.79	0.41	7.57	0.10	**0.0000**
Epsilonbacteraeota	*Campylobacteraceae*	5.29	2.67	5.23	2.85	0.6857
Firmicutes	*Acidaminococcaceae*	7.15	0.18	7.45	0.14	**0.0009**
	Bacillaceae	5.92	0.15	<LOQ	2.84	**0.0019**
	Christensenellaceae	<LOQ	0.00	6.37	0.16	**0.0000**
	Clostridiaceae_1	5.96	0.59	5.17	2.64	**0.0333**
	Defluviitaleaceae	<LOQ	0.00	6.10	0.29	**0.0000**
	DTU014_fa	<LOQ	0.00	5.69	2.54	**0.0001**
	Enterococcaceae	6.44	3.18	6.47	3.18	0.9890
	Erysipelotrichaceae	<LOQ	0.00	6.12	0.22	**0.0000**
	Eubacteriaceae	<LOQ	0.00	6.75	0.13	**0.0000**
	Family_XIII	<LOQ	0.00	6.67	0.16	**0.0000**
	Firmicutes_unclassified	5.13	2.79	5.30	2.86	0.9607
	Lachnospiraceae	8.38	0.17	8.79	0.07	**0.0000**
	Paenibacillaceae	5.55	3.00	<LOQ	2.68	0.8769
	Planococcaceae	6.11	0.22	5.44	1.83	0.0621
	Ruminococcaceae	6.63	0.33	7.68	0.11	**0.0000**
	Veillonellaceae	9.16	0.04	9.20	0.10	0.3927
Lentisphaerae	*vadinBE97*	<LOQ	0.00	6.50	0.21	**0.0000**
	Victivallaceae	<LOQ	0.00	6.59	0.34	**0.0000**
Proteobacteria	*Burkholderiaceae*	6.60	0.36	6.81	0.15	**0.0373**
	Desulfovibrionaceae	6.27	2.66	7.67	0.13	**0.0030**
	Enterobacteriaceae	7.91	0.54	7.23	0.42	**0.0312**
	Pseudomonadaceae	7.12	0.68	7.16	0.26	0.0572
	uncultured	6.41	3.41	6.19	2.04	0.0848
Synergistetes	*Synergistaceae*	<LOQ	0.00	5.38	2.99	**0.0225**
Verrucomicrobia	*Akkermansiaceae*	<LOQ	0.00	6.45	0.60	**0.0000**
	Puniceicoccaceae	<LOQ	0.00	6.01	0.38	**0.0000**
