# Supplementary material for: Co-Supplementation of Baobab Fiber and Arabic Gum Synergistically Modulates the In Vitro Human Gut Microbiome Revealing Complementary and Promising Prebiotic Properties
Source: Nutrients. 2024 May 22;16(11):1570. doi: 10.3390/nu16111570 (PMC11173755; doi:10.3390/nu16111570)
Supplement: Supplementary file 1 [file nutrients-16-01570-s001.zip › Supplementary Table S3.pdf]

Table S3: Treatment effect on the microbial community composition at OTU level during the long-term SHIME® experiment. Relative levels (log (cells/mL)) of the 25 most abundant OTUs, belonging to specific phyla and families, observed in the proximal colon (PC) and distal colon (DC) vessels upon treatment with baobab fiber (BF), arabic gum (AG), and the combination of both (BF + AG). The closely related species, as identified by blasting of the corresponding sequence, were also included for each OTU. Relative values were obtained by subtracting the average levels obtained at the end of the control period (n = 3) from the corresponding levels obtained at the end of the treatment period (n=3). The intensity of the shading correlates with the relative abundance, normalized for each of the different OTUs (i.e., within each row). Statistically significant differences between the absolute levels at the end of the control period and the absolute levels at the end of the treatment period were indicated by means of '*' ($p<0.05$).

Phylum	Family	OTU	Closely related species	PC			DC		
				BF	AG	BF + AG	BF	AG	BF + AG
**Actinobacteria**	***Bifidobacteriaceae***	**Otu00002**	***Bifidobacterium adolescentis***	0.67*	-0.64*	0.66*	0.45*	-0.76*	0.02
		**Otu00006**	***Bifidobacterium longum***	-0.34*	1.00*	0.01	-0.16	0.97*	0.17
	Microbacteriaceae	**Otu00017**	***Microbacterium sp.***	0.84	-0.93	-0.76	0.47	-0.87	-0.74*
**Bacteroidetes**	***Bacteroidaceae***	**Otu00004**	***Bacteroides fragilis***	-1.94*	-1.32*	-1.44*	-1.40*	-1.56*	-1.07*
		**Otu00005**	***Bacteroides vulgatus***	1.46*	<LOQ	2.05	1.10*	0.60*	1.00*
		**Otu00007**	***Bacteroides ovatus***	-0.63*	-0.87*	0.11	0.48*	-0.02	-0.10
		**Otu00009**	***Bacteroides cellulosilyticus***	-1.37*	<LOQ	<LOQ	0.20	0.78*	-0.13
		**Otu00015**	***Bacteroides thetaiotaomicron***	-1.37*	<LOQ	-1.36*	0.92*	0.19	0.71*
		**Otu00021**	***Bacteroides uniformis***	0.61	<LOQ	-0.74	0.58*	0.36*	0.48*
	Prevotellaceae	**Otu00011**	***Prevotella sp.***	1.41*	2.96*	1.33*	0.68	1.62*	0.78
**Firmicutes**	***Lachnospiraceae***	**Otu00003**	***Clostridium clostridioforme/bolteae***	-0.27	-0.15	-0.19	-0.12	-0.21	-0.31*
		**Otu00010**	***Clostridium sp.***	0.93*	1.35*	1.16*	0.67*	1.30*	0.68
		**Otu00008**	***Eubacterium rectale***	-0.62*	-0.02	-0.63*	-0.29	-0.27*	-0.73*
		**Otu00020**	***Blautia sp.***	-0.42*	1.54*	0.70*	0.29	1.56*	0.94*
		**Otu00039**	***Clostridium lavalense***	<LOQ	<LOQ	<LOQ	0.58*	1.50*	0.20*
		**Otu00022**	***Ruminococcus torques***	<LOQ	<LOQ	<LOQ	0.16	-0.66*	-0.34*
		**Otu00035**	***Lachnospiraceae spp.***	<LOQ	<LOQ	<LOQ	0.78*	1.48*	1.34*
		**Otu00018**	***Roseburia faecis***	0.78*	1.61*	1.30*	0.92*	0.98*	0.77*
		**Otu00030**	***Lachnospiraceae spp.***	-0.77*	1.44*	0.41	-0.43*	0.96*	0.54
		**Otu00014**	***Eisenbergiella sp.***	<LOQ	0.46*	<LOQ	0.24	0.13*	0.27*
	Ruminococcaceae	**Otu00019**	***Gemmiger formicilis***	-0.60	<LOQ	<LOQ	0.23	2.84*	2.57*

		**Otu00013**	***Faecalibacterium prausnitzii***	0.94	0.78*	1.29	1.62*	0.61	1.17
	Veillonellaceae	**Otu00001**	***Megamonas sp.***	-0.09	-0.17	-0.22*	-0.39	-0.15*	-0.03
**Proteobacteria**	***Enterobacteriaceae***	**Otu00012**	***Klebsiella oxytoca***	0.06	-0.68	-0.58	0.25	-1.02	0.40
		**Otu00029**	***Klebsiella oxytoca***	0.28	-1.13	-0.58	0.38	-1.11*	0.43
